# Shifting interactions among bacteria, fungi and archaea enhance removal of antibiotics and antibiotic resistance genes in the soil bioelectrochemical remediation

**DOI:** 10.1186/s13068-019-1500-1

**Published:** 2019-06-24

**Authors:** Xiaodong Zhao, Xiaojing Li, Yue Li, Yang Sun, Xiaolin Zhang, Liping Weng, Tianzhi Ren, Yongtao Li

**Affiliations:** 1 Agro-Environmental Protection Institute, Ministry of Agriculture and Rural Affairs, Key Laboratory of Original Agro-Environmental Pollution Prevention and Control, MARA/Tianjin Key Laboratory of Agro-Environment and Agro-Product Safety, Tianjin, 300191 China; 2 College of Natural Resources and Environment, South China Agricultural University, Guangzhou, 510642 China

**Keywords:** Soil bioelectrochemical remediation, Bioelectricity generation, Antibiotic degradation, Antibiotic resistance genes, Biological interactions

## Abstract

**Background:**

Antibiotics and antibiotic resistance genes (ARGs) are two pollutants in soil, especially ARGs as one of the top three threats to human health. The performance of soil microbial fuel cells (MFCs) fuelled with antibiotics was investigated.

**Results:**

In this study, soil MFCs spiked with tetracycline exhibited optimal bioelectricity generation, which was 25% and 733% higher than those of MFCs spiked with sulfadiazine and control, respectively. Compared with the non-electrode treatment, not only did functional micro-organisms change in open- and closed-circuit treatments, but also the microbial affinities, respectively, increased by 50% and 340% to adapt to higher removal of antibiotics. For the open-circuit treatment, the ineffective interspecific relation of micro-organisms was reduced to assist the removal efficiency of antibiotics by 7–27%. For the closed-circuit treatment, an intensive metabolic network capable of bioelectricity generation, degradation and nitrogen transformation was established, which led to 10–35% higher removal of antibiotics. Importantly, the abundances of ARGs and mobile genetic element (MGE) genes decreased after the introduction of electrodes; especially in the closed-circuit treatment, the highest reduction of 47% and 53% was observed, respectively.

**Conclusions:**

Soil MFCs possess advantages for the elimination of antibiotics and ARGs with sevenfold to eightfold higher electricity generation than that of the control treatment. Compared with sulphonamides, the enhancement removal of tetracycline is higher, while both potential ARG propagation risk is reduced in soil MFCs. This study firstly synchronously reveals the relationships among bacteria, fungi and archaea and with ARGs and MGE genes in soil bioelectrochemical systems.
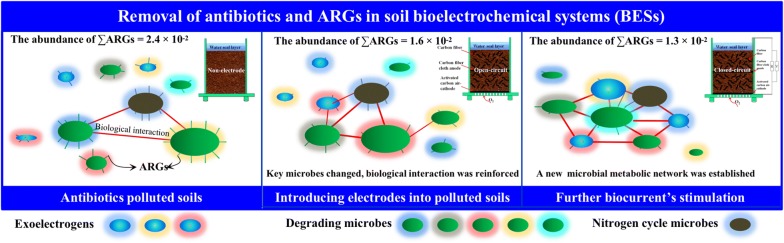

**Electronic supplementary material:**

The online version of this article (10.1186/s13068-019-1500-1) contains supplementary material, which is available to authorized users.

## Background

A recent research showed that total antibiotic usage reached 92,700 tons in China, of which veterinary antibiotics accounted for up to 52% [1]. Antibiotics and its metabolites are excreted into water, sediments and soils in the faeces or urine (excretion rates range from 40 to 90%) [2, 3]. In fact, approximately 84% of the total excretion of antibiotics (54,000 tons) stemmed from livestock industries in China [1]. Unfortunately, residual antibiotics are transferred into agricultural soils as soon as animal wastes are used as fertilizer [4]. For example, sulphonamides and tetracyclines are commonly detected in agricultural soils at concentrations of 6–33 and 5–25 mg kg^−1^, respectively [5]. In the presence of antibiotics, some microbial genes may mutate (or the gene expression change), which lead to the development of antibiotic resistance [6]. The horizontal gene transfer of antibiotic resistance genes (ARGs) via mobile genetic elements is an immeasurable threat to humans [7]. Therefore, developing a removal method for antibiotics and ARGs in soil is increasingly desirable.

Biodegradation is a low-cost, eco-friendly method for the removal of organic contamination from soils, but electron acceptor deficiency restricts the sustainability of this technology [8]. Promisingly, soil microbial fuel cells (MFCs) represent an emerging remediation technology that provides an inexhaustible electron acceptor and simultaneously employs micro-organisms as catalysts to directly convert chemical energy into electricity [9, 10]. Soil MFC technology has shown excellent effectiveness in the degradation of polycyclic aromatic hydrocarbons [11], petroleum hydrocarbon [12, 13], phenol [14] and pesticides [15] in soils. Biocurrent generated from soil MFCs can stimulate the growth of functional microbial populations and thus improve their biodegradation efficiency [13, 16]. However, the tremendous internal resistance of soil limits electron transfer and the further improvement of soil MFC performance [8]. As an amendment for soil MFCs, mixing conductive carbon fibre into soils reduced the internal resistance by 58% and increased the degradation rates of petroleum hydrocarbons by 100–329% [17]. Recently, MFC technology has performed well in degrading antibiotics (e.g. after 10 months of domestication, 85% of 20 mg L^−1^ of sulfamethoxazole was removed within 12 h) and inhibiting ARGs in wastewater [18–22], but little has been reported concerning its effect on polluted soils.

During the process of biodegradation, functional micro-organisms (e.g. exoelectrogens and degraders) cannot operate alone and a complicated community network architecture is present in their microworld. For example, *Anaerolineaceae* fermentative bacteria break down small-molecule saccharides into short-chain fatty acids and H_2_, thus providing electron donors for electrochemically active bacteria such as *Geobacteraceae* and denitrifying bacteria such as *Rhodocyclaceae* and *Comamonadaceae* [23]. Additionally, homoacetogens were found to boost the conversion rate of acetate in MFCs by providing substances for exoelectrogens and methanogens, even these synergistic interactions occurred among fermentative bacteria, homoacetogens, exoelectrogens and methanogens [24, 25]. To date, few studies have detailed the community structures and interspecific relationships among bacteria, fungi and archaea in MFC systems concurrently.

In this study, tetracycline and sulfadiazine, which are commonly detected in soils, were selected as typical antibiotics to study in soil MFCs, with the following aims: first, to investigate the ability of soil MFCs to degrade tetracycline and sulfadiazine and to generate electricity by constructing microbial electrochemical systems; second, to explore whether soil MFCs can inhibit the occurrence of ARGs and reduce their propagation by using a Smartchip real-time PCR system; and last, to integrally reveal the change of structures and interactions of the bacterial, fungal and archaeal communities after the introduction of electrodes and further stimulation of biocurrent by 16S rRNA sequencing.

## Results

### Bioelectricity generation by the soil MFCs

**Fig. 1:**
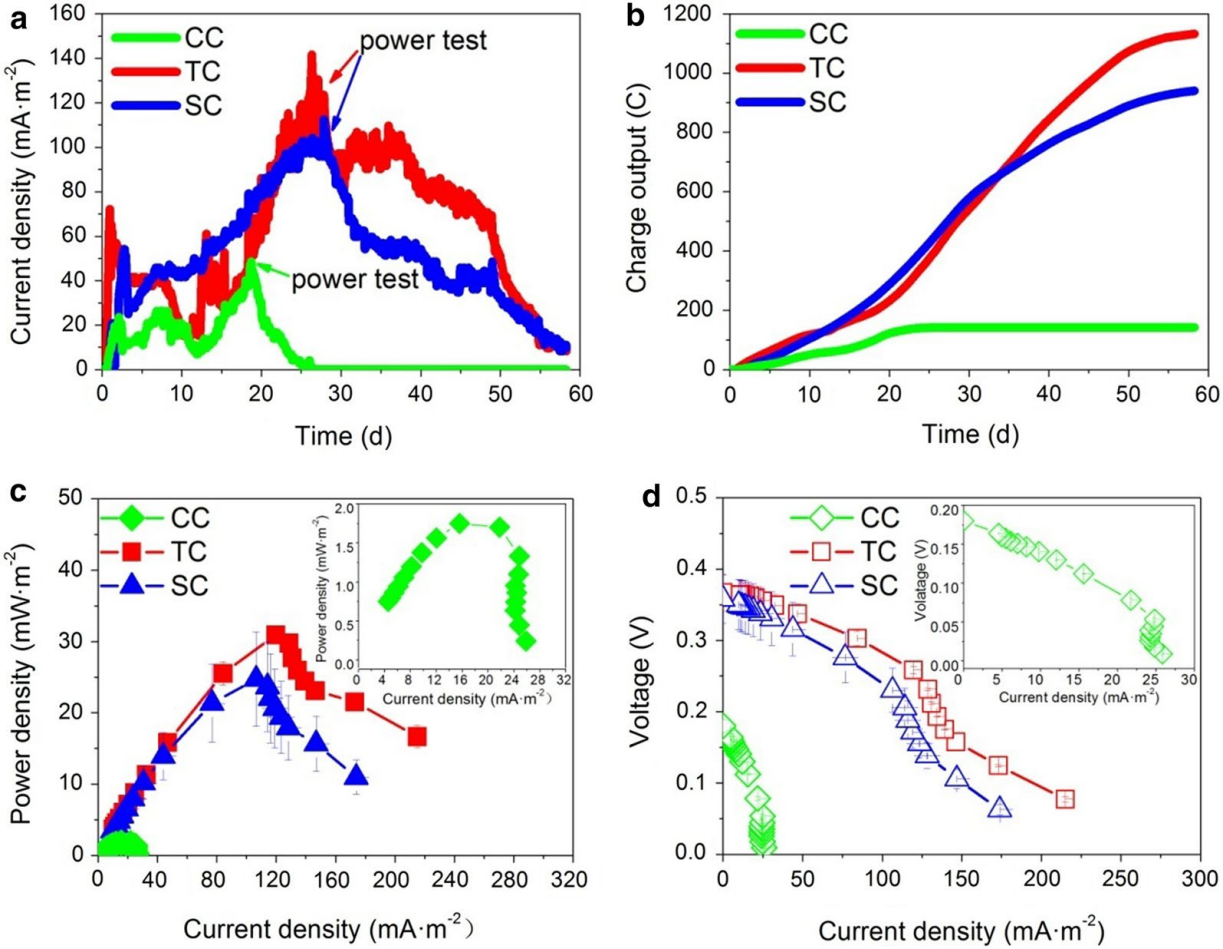
Current density (**a**) and charge output (**b**) over an incubation time of 58 days, and power density (**c**) and polarization (**d**) curves of the soil MFCs. Closed-circuit treatments spiked with tetracycline, sulfadiazine and without antibiotics added are marked as TC, SC and CC, respectively. Data are the means of duplicates

The start-up time for the soil MFCs was defined as the time needed for the voltage output to reach greater than 1 mV (100 Ω of external resistance). The start-up time of TC (closed-circuit treatments spiked with tetracycline) was 9 h (Fig. 1a and Additional file 1: Figure S1a), which was 7–8 h earlier than those of SC (closed-circuit treatments spiked with sulfadiazine, 16 h) and CC (closed-circuit treatments without the addition of antibiotics, 17 h). Within 1–3 days, the first peak of current density was 65 ± 6 mA m^−2^ for TC, which was 23% and 196% higher than those for SC (53 ± 1 mA m^−2^) and CC (22 ± 1 mA m^−2^), respectively. On the 27th day, the maximum current densities of TC and SC achieved 136 ± 3 and 109 ± 6 mA m^−2^, respectively, while that of CC was only 47 ± 1 mA m^−2^ on day 19. The accumulated charge output was 1132 ± 48 C for TC and 940 ± 82 C for SC, which were sevenfold to eightfold more than that of CC (142 ± 26 C) (Fig. 1b). Power density and polarization curves were analysed when the soil MFCs reached their maximum current densities (Fig. 1c, d), which were as follows: TC (31 ± 1 mW m^−2^) > SC (25 ± 7 mW m^−2^) > CC (1.8 ± 0.03 mW m^−2^). Furthermore, the open-circuit voltages (OCVs) of TC, SC and CC were 0.37 ± 0.02, 0.36 ± 0.03 and 0.18 ± 0.01 V, respectively (Additional file 1: Figure S1b). In terms of their individual polarization curves, the internal resistances of SC and TC were 336 and 262 Ω, respectively, 75–80% lower than that of CC (1332 Ω).

### Removal of tetracycline and sulfadiazine

**Fig. 2:**
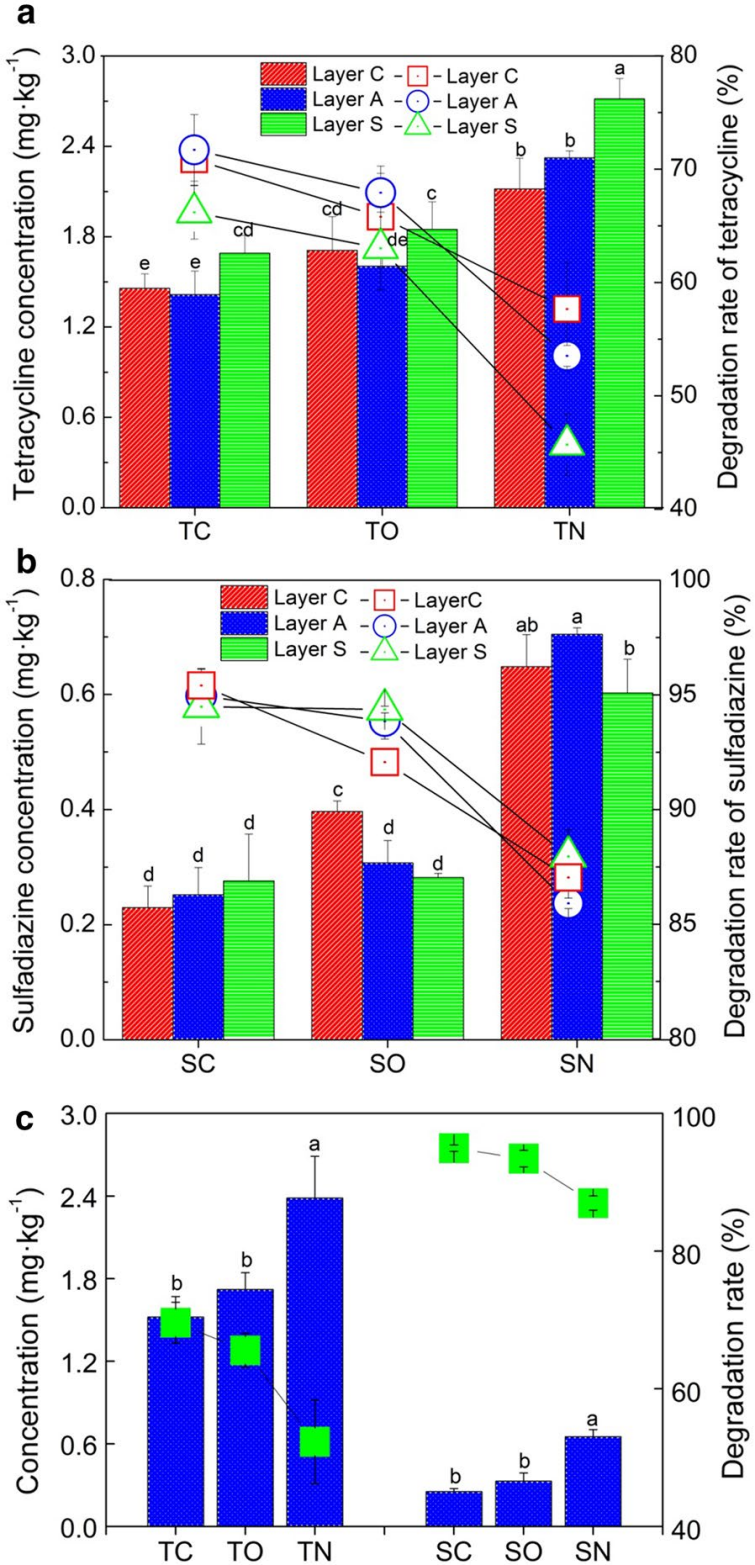
Concentrations (histogram) and degradation rates (line chart) of tetracycline (**a**) and sulfadiazine (**b**) in different layers of soil MFCs. The average concentrations and degradation rates of antibiotics (**c**). Different lowercase letters represent significant differences at the 0.05 level among TC, TO and TN or SC, SO and SN, respectively. The closed-circuit, open-circuit and non-electrode treatments spiked with tetracycline were, respectively, labelled as TC, TO and TN; those spiked with sulfadiazine were, respectively, labelled as SC, SO and SN

After 58 days of incubation, the removal efficiencies of tetracycline and sulfadiazine increased from 46 ± 2.7 to 72 ± 3.1% and 86 ± 0.2 to 95 ± 0.7% in closed-circuit MFCs compared with the corresponding non-electrode controls, respectively (Fig. 2a, b). As expected, the average degradation rate of TC exhibited the highest value (70 ± 3%), followed by those of TO (open-circuit treatments spiked with tetracycline, 66 ± 2.5%) and TN (non-electrode treatments spiked with tetracycline, 52 ± 6.1%) (Fig. 2c). Meanwhile, the degradation rates of sulfadiazine showed the same trend as those of tetracycline: SC (95 ± 0.5%) > SO (open-circuit treatments spiked with sulfadiazine, 93 ± 1.2%) > SN (non-electrode treatments spiked with sulfadiazine, 87 ± 1%). Unexpectedly, the introduction of electrodes (open circuit) promoted the degradation of antibiotics, at rates 7–27% higher than those of corresponding non-electrode controls (*p *< 0.05, Duncan’s test). The concentrations in layer C of the closed circuits were 15–42% lower than their corresponding layers in the open circuits (Fig. 2a, b, *p *< 0.05, Duncan’s test). The lowest residual concentration of TC occurred in layer A (1.4 ± 0.2 mg kg^−1^) and a slight increase was found in layer C (1.5 ± 0.1 mg kg^−1^), both of which were 14–16% lower than that in layer S (Fig. 2a, *p *< 0.05, Duncan’s test). For SC, the lowest sulfadiazine residue was observed in layer C (0.23 ± 0.04 mg kg^−1^), which was 9–17% lower than those in the other layers (Fig. 2b).

### Evolution of target genes

**Fig. 3:**
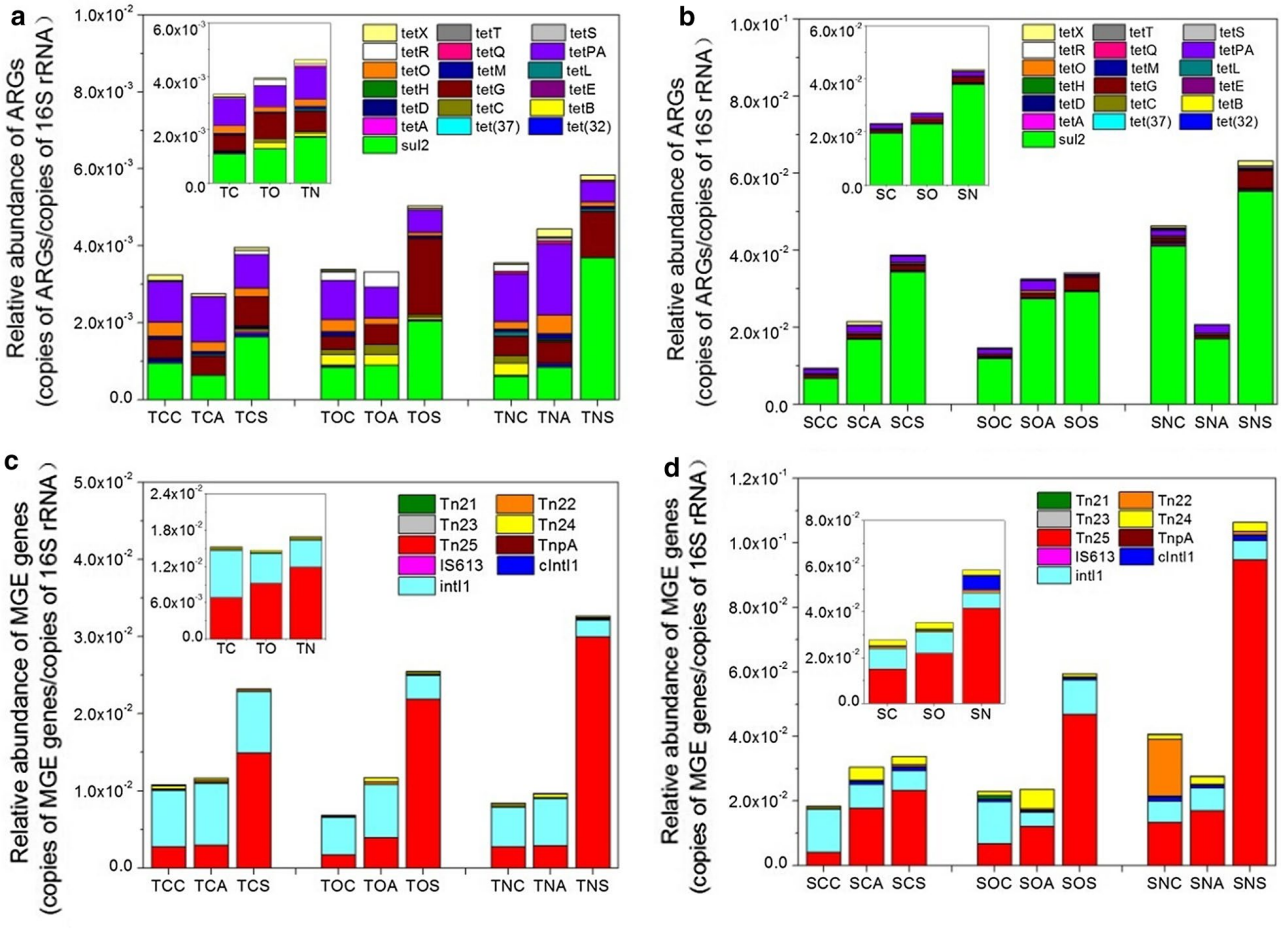
Relative abundances of *tet* and *sul* genes in treatments spiked with tetracycline (**a**) and sulfadiazine (**b**). Relative abundances of MGE genes in treatments spiked with tetracycline (**c**) and sulfadiazine (**d**). TC/TO/TN/SC/SO/SNC, TC/TO/TN/SC/SO/SNA and TC/TO/TN/SC/SO/SNS represent layer C, layer A and layer S in the same rector, respectively

In this study, thirty-seven target genes including twenty-eight antibiotic resistance genes and nine mobile genetic element genes were detected (Additional file 1: Table S1). Eighteen *tet* genes were found in the tested soils, and the detection rates of *tet*G and *tet*PA were 100% (*n *= 27) (Additional file 1: Figure S2). Among the *sul* genes, only the *sul*2 gene was detected, with a 100% detection rate (*n *= 27). All MGE genes were detected and the predominant genes were *intI*1, *Tn*24 and *Tn*25, which had detection rates of 100% (*n *= 27). The relative abundances of the target genes were normalized to the 16S rRNA gene (copies of gene/copies of 16S rRNA). The introduction of electrodes (open circuit) reduced the abundances of ARGs and MGE genes, which further declined under biocurrent stimulation (closed circuit), except for MGE genes in TC (Fig. 3). For example, compared with SN, these genes declined by approximately 39% in SO and showed a 14–22% decrease again in SC. Meanwhile, the abundances of ARGs and MGE genes in C and A layers were lower than those in corresponding S layers, especially for TC, which showed decreases of 18–54%. The least abundances of ARGs occurred in layer A of TC and layer C of the SC, consistent with the residual antibiotic concentrations in TC and SC.

### Microbial communities

A total of 73,327–95,166, 52,654–95,683 and 75,515–94,890 effective tags with average lengths of 253, 221 and 280 bp were acquired for the bacterial, fungal and archaeal communities, respectively, through 16S rRNA amplicon sequencing of soil samples; furthermore, 1757–5392, 400–859 and 127–1006 operational taxonomic units (OTUs) at a 97% similarity clustered, respectively (Additional file 1: Table S2). Meanwhile, the Good’s coverage estimators were all above 98%, indicating that the sequencing depths were adequate for the microbial communities.

#### Bacterial community

The Shannon and Chao1 indices of the bacterial community were more sensitive to the addition of sulfadiazine than that of tetracycline (Additional file 1: Table S3). Compared with those of CC, the SC indices exhibited decreases of 9–25%. Meanwhile, the SC layer S indices decreased by 28–50% compared to those of SN (Additional file 1: Table S4).

**Fig. 4:**
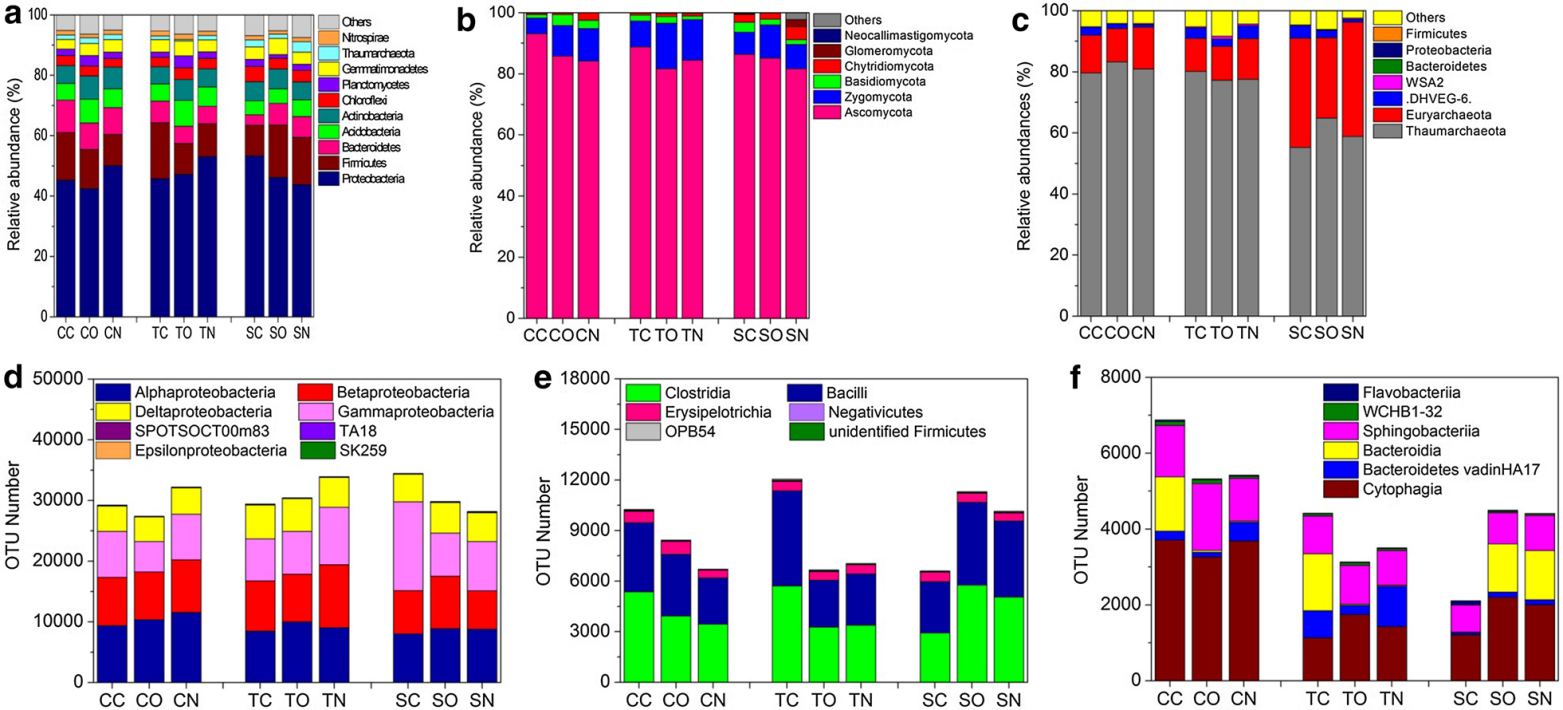
Taxonomic classification of the microbial DNA sequences from soil communities in the MFCs at the phylum level for bacteria (**a**), fungi (**b**), and archaea (**c**); those at the class level distribution of the dominant bacterial phyla of *Proteobacteria* (**d**), *Firmicutes* (**e**), *Bacteroides* (**f**). CC represents the closed-circuit treatment without antibiotics added; CO and CN, respectively, represent the corresponding open-circuit and non-electrode treatments

*Proteobacteria*, *Firmicutes* and *Bacteroides* were the top three phyla, and their abundances accounted for 52–84% of the total bacterial community in all samples (Fig. 4a). Compared with the corresponding non-antibiotic treatments, the OTUs of *Proteobacteria* increased up to 18% with antibiotic addition, while *Bacteroides* decreased by 19–69% (Fig. 4d–f). Compared with SN, the abundance of *Proteobacteria* increased by 5% in SO and further increased by 16% in SC. The introduction of electrodes slightly decreased the abundances of *Firmicutes* and *Bacteroides* in treatments spiked with tetracycline (3–6%), whereas obvious increases of 28–81% were observed under biocurrent stimulation. Higher abundances of *Betaproteobacteria* and *Gammaproteobacteria* were observed in layer S in all treatments, whereas the abundance of *Deltaproteobacteria* in layers A and C was 47–212% and 56–388% higher than that in layer S, respectively (Additional file 1: Figure S3b). Species of *Firmicutes* (mainly *Clostridia* and *Bacillus*, 89–96%) clustered in layer A, such as in SC, where 227–364% of growth was viewed in layer A compared to the other layers (Additional file 1: Figure S3c). *Bacteroides* was primarily found in layer A, except for in SC and SO (Additional file 1: Figure S3d).

**Fig. 5:**
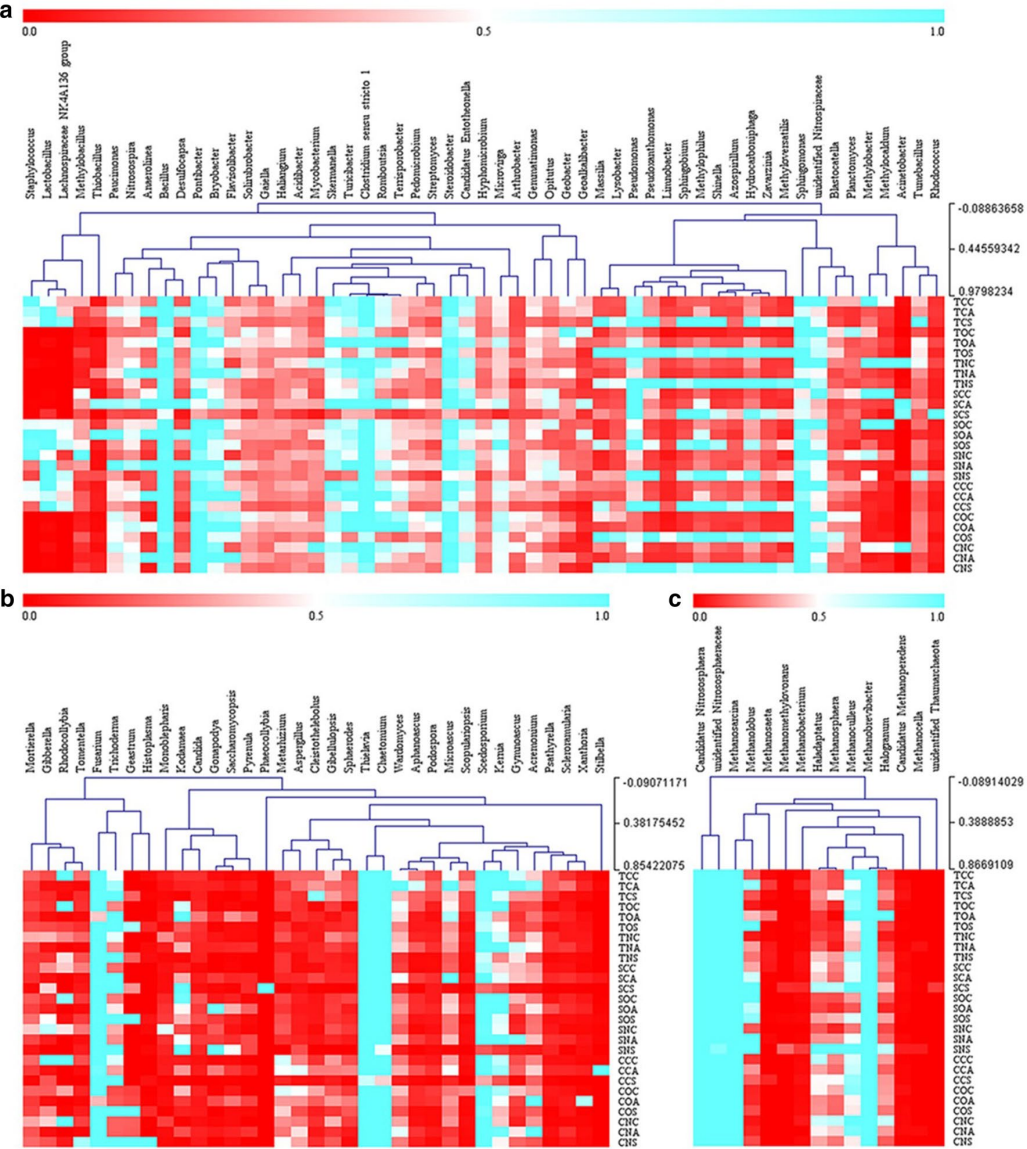
Taxonomic classification of microbial DNA sequences from soil communities in different layers of MFCs at the genus level of bacteria (**a**), fungi (**b**), archaea (**c**)

The distribution of the top 55 genera accounted for 20–54% of the total bacterial composition (Fig. 5a and Additional file 1: Figure S4a). The top one genus was *Pseudomonas* (3%, *Proteobacteria*); compared with the corresponding non-antibiotic treatments, the abundance of *Pseudomonas* increased by 13–155% and 160–691% after the addition of tetracycline and sulfadiazine, respectively. The amounts of *Methylobacter*, *Desulfocapsa* and *Geoalkalibacter* rose, respectively, by 92–666%, 29–299% and 42–376% after the addition of antibiotics. Those of *Tumebacillus*, *Geobacter*, *Acinetobacter* and *Pseudoxanthomonas*, respectively, increased by 46–281%, 91–229%, 13–736% and 37–175% after the addition of tetracycline, while the abundances of *Anaerolinea* and *Methylobacillus*, respectively, increased by 13–224% and 120–557% with the addition of sulfadiazine. Compared with the corresponding non-electrode treatments, after the introduction of electrodes, the abundances of *Pseudoxanthomonas* and *Geobacter*, respectively, increased by 45% and 103% in treatments spiked with tetracycline. Relative to the corresponding open-circuit treatments, the amounts of *Anaerolinea*, *Desulfocapsa* and *Tumebacillus* increased by 42–60%, 58–69% and 24–106% after the stimulation of biocurrent, respectively. In the connected soil MFCs, the amount of *Bacillus* in layer A was 16–452% higher than that in other layers of the same reactor (Fig. 5a). Species of *Desulfocapsa* showed similar results, with 13- to 14-fold higher increases in SC. In treatments spiked with tetracycline, the amounts of *Geobacter* and *Geoalkalibacter* in layers C and A increased, respectively, by 63–285% and 132–645% compared to those in layer S of the closed- or open-circuit groups.

#### Fungal community

Compared with the corresponding non-antibiotic treatments, the Shannon (except for TN) and Chao1 (except for SC) indices increased after the addition of antibiotics, especially for TC (18–24% of increase) (Additional file 1: Table S3). Compared with the corresponding non-electrode treatments, the Shannon index declined by 10% and 4% with the introduction of electrodes in treatments spiked with sulfadiazine and in the non-antibiotic treatments, respectively. The index further decreased by 6–11% under biocurrent stimulation. An opposite trend was observed for the Shannon index in treatments spiked with tetracycline.

At the phylum level, the abundance of *Ascomycota* accounted for 82–93% of the total composition of all libraries, followed by *Zygomycota* (8–15%), *Basidiomycota* (1–4%) and *Chytridiomycota* (0.5–2.6%) (Fig. 4b). Compared with the corresponding non-antibiotic treatments, the OTUs of *Ascomycota* decreased after the addition of antibiotics except for TN, with the highest value being 3656 OTUs. However, those of *Zygomycota* and *Chytridiomycota* increased by 8–68% (except for SN) and 51–463% (except for TN), respectively. Compared with the corresponding non-electrode treatments, the OTUs of *Ascomycota* rose by 816–1858 OTUs in CO (open-circuit treatments without the addition of antibiotics) and SO and increased again by 596–3840 OTUs under biocurrent stimulation. Species of *Zygomycota* were more abundant in layer A than other layers (Additional file 1: Fig. S5b). In treatments spiked with sulfadiazine, the abundance of *Zygomycota* in layer A decreased by 16% with the introduction of electrodes compared to the non-electrode treatment, but it increased by 29% under biocurrent stimulation.

The abundances of the top 35 genera were selected to cluster the heat map and accounted for the majority of the total composition (Fig. 5b and Additional file 1: Figure S4b). The top two genera were *Fusarium* (11%) and *Trichoderma* (5%). *Trichoderma* abundance rose by 64–670% and 49–191% with tetracycline and sulfadiazine added, respectively. Compared with the corresponding non-electrode treatments, after the introduction of electrodes, the amount of *Trichoderma* increased by 149% in treatments spiked with sulfadiazine, and it further rose by 82% by biocurrent stimulation. The amount of *Kernia* rose by 19–102% with the addition of antibiotics (except for SC). In connected soil MFCs, compared with the corresponding non-antibiotic treatments, the abundances of *Wardomyces* and *Gymnoascus*, respectively, increased by 14–234% and 114–506% by antibiotics added. The abundance of *Wardomyces* exhibited the increments of 23–148% in both open- and closed-circuit treatments spiked with antibiotics compared with the corresponding non-electrode treatments. Furthermore, the abundance of *Microascus* was stimulated by the addition of antibiotics and exhibited the largest increase in the closed-circuit groups (93–122%), followed by the open-circuit groups (32–97%) and non-electrode groups (12–50%). The amount of *Microascus* increased by 44–90% with the introduction of electrodes compared with the corresponding non-electrode treatments and further rose by 17–71% under biocurrent stimulation. Strikingly, the abundance of *Microascus* was predominant in layer A of the connected soil MFCs, with its highest increase being 55 times that in other layers of the same reactor (Fig. 5b).

#### Archaeal community

Compared with the corresponding non-antibiotic treatments, the Chao1 index rose by 48–84% in treatments spiked with tetracycline (Additional file 1: Table S3). Compared with the corresponding non-electrode treatments, the Chao1 index increased by 12–31% with the introduction of electrodes and further increased by 4–21% under biocurrent stimulation in treatments spiked with antibiotics. The fluctuations of the Chao1 index in layered soils were more obvious than those of the Shannon index after the addition of tetracycline, especially in layer A of TC, where the Chao1 index rose by 183% (Additional file 1: Table S4).

*Thaumarchaeota* and *Euryarchaeota* were the dominant archaea in soil MFCs and accounted for 88–96% of the total composition in all libraries (Fig. 4c). Compared with the corresponding non-antibiotic treatments, species of *Thaumarchaeota* and *Euryarchaeota* were more sensitive to the addition of sulfadiazine. Consequently, 22–31% decreases were observed for *Thaumarchaeota*, while increases of 140–189% were found for *Euryarchaeota*. Compared with the corresponding non-electrode treatments, the amount of *Euryarchaeota* in layer A declined by 18% with the introduction of electrodes in treatments spiked with tetracycline (Additional file 1: Figure S6a); it further decreased by 47% under biocurrent stimulation. Opposite trends were seen in the treatments spiked with sulfadiazine.

The top 15 genera accounted for 31–91% of the total composition (Fig. 5c and Additional file 1: Figure S4c). *Candidatus Nitrososphaera* (16%, *Thaumarchaeota*), *Methanosarcina* (12%, *Euryarchaeota*) and unidentified *Nitrososphaeraceae* (11%, *Thaumarchaeota*) were the dominant genera of archaea in soil MFCs. Compared with the corresponding non-antibiotic treatments, the amount of *Candidatus Nitrososphaera* increased after the addition of tetracycline, with a highest value of 28%; however, it fell by 15–25% after the addition of sulfadiazine. Opposite trends were observed for *Methanosarcina* (except for TN). *Methanosarcina* abundance exhibited an increase of 16% in SO compared with SN, which further increased by 10% in SC. Notably, the genus *Methanosarcina* was predominant in layer A of SC, with an abundance 420% higher than that in TC (Fig. 5c); meanwhile, it declined by 72% in layer A of TC compared with that of TN.

## Discussion

The bioelectricity generation of CC was better than that of our previous work (without carbon fibre in the soil). For instance, the maximum current density and charge output of CC were, respectively, 5.7- and 3.8-fold higher than those of our previous report [26] (Additional file 1: Figure S7). Mixing carbon fibre into soil in MFCs reduced internal resistance and simultaneously promoted the electron transfer rate to the anode [17], which means that electron donors such as soil organic matters were efficiently utilized. The electricity generation of TC and SC was obviously superior to that of CC, suggesting that soil MFC systems can directly convert antibiotics into electricity in soils, especially for tetracycline (Fig. 1b).

Previous studies have shown that tetracycline is difficult to biodegrade, whereas sulphonamides have been found to be easily assimilated as a carbon or nitrogen source for microbial growth [27, 28]. However, Wang et al. [29] stressed the essentiality of the gradual domestication of micro-organisms during the process of tetracycline removal by MFCs, and 79% of tetracycline was removed within 7 days in closed MFCs after a domestication cycle of 5 weeks. Here, no inoculation and acclimation resulted in a relatively slow tetracycline degradation efficiency. Sulfadiazine is firstly biodegraded to hydroxyl sulfadiazine and dihydroxyl sulfadiazine; these products can be further degraded to *p*-aminobenzenesulfonic acid and 2-amino-4,6-dihydroxypyrimidine [22, 30]. Interestingly, a mono-oxygenation reaction is crucial for the first two steps of sulfadiazine biodegradation, which requires electron donors and molecular oxygen [30]. Coincidentally, such an environment appeared near the air–cathode, which accelerated sulfadiazine biodegradation near the cathodic soils. Unexpectedly, the degradation rates of the antibiotics in TC and SC increased only by 2–6% over those in TO and SO, respectively. A similar phenomenon was found for the targeted genes. These results suggest that there is a measurable increase in efficiency in terms of antibiotic removal and ARG control as long as the open-circuit condition in the soil MFCs is maintained. There are two possible reasons for this phenomenon: on the one hand, conductive materials can promote direct interspecies electron transfer between microbial species, thus enhancing antibiotic degradation [31]; on the other hand, the introduction of electrodes likely forms a self-induced circuit where the generated electrons are consumed in situ with the help of electron acceptors such as oxygen, nitrate and sulphate [16, 17].

**Fig. 6:**
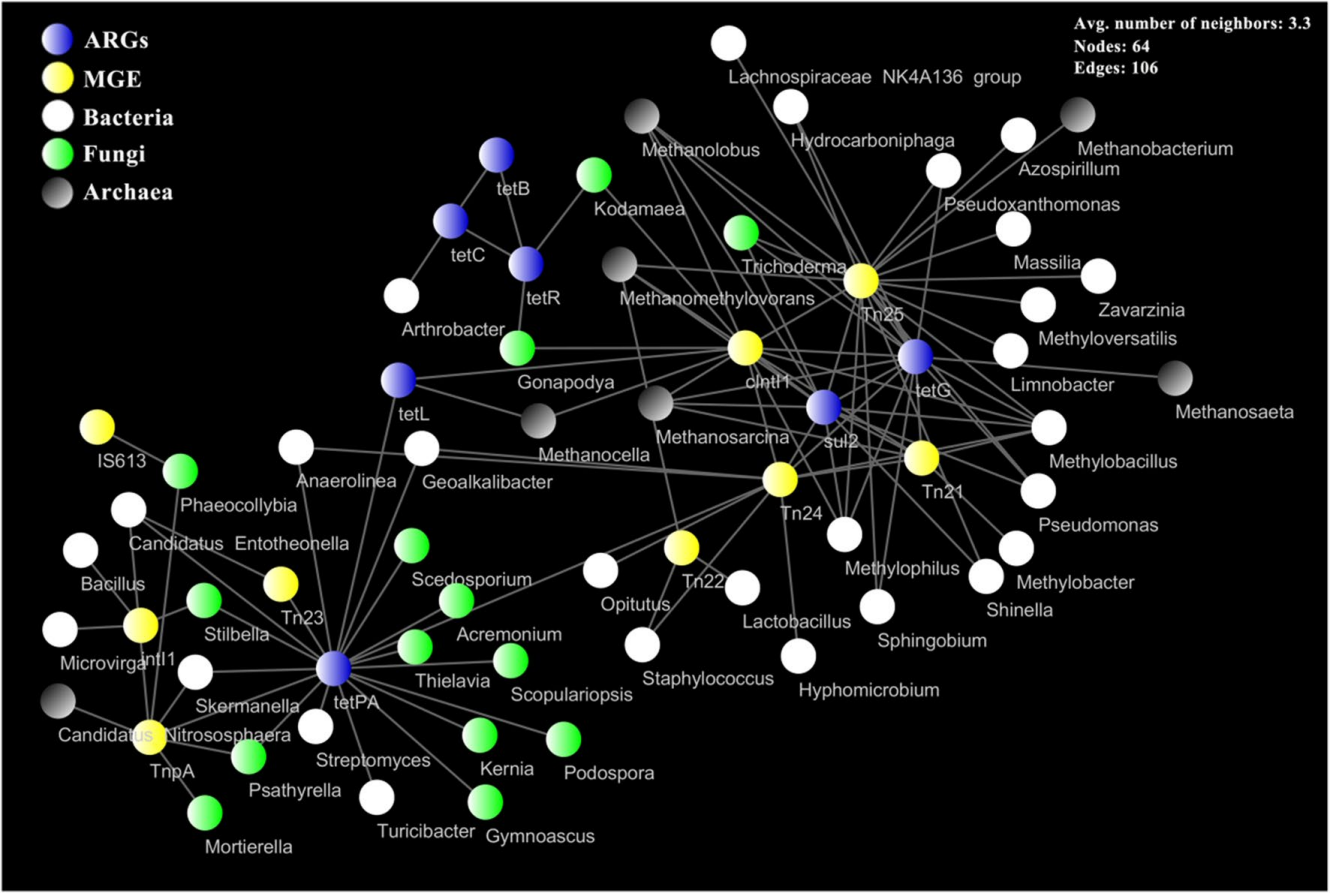
Network analysis showing the connectedness among ARGs, MGE genes and micro-organisms at genus level. Each edge stands for significant the positive correlation between two items (*p* < 0.05, Spearman test)

The network analysis of ARGs, MGE genes and micro-organisms was performed to reveal the potential hosts and possible propagation ways of ARGs (Fig. 6). In the present research, *tet*PA gene had the broadest host range, and 65% of those belonged to fungi. *Tnp*A and *Tn*23 genes had intense links with *tet*PA gene. However, it lacked close links among *Tnp*A, *Tn*23 genes and the potential hosts of *tet*PA gene. *Sul*2 and *tet*G shared the same hosts with the genera of *Pseudomonas*, *Methylophilus*, *Methylobacillus* (bacteria); *Trichoderma* (fungus); *Methanosarcina*, *Methanolobus* and *Methanomethylovorans* (archaea). Meanwhile, close links were found among *sul*2, *tet*G, *clntl*1, *Tn*21, *Tn*24 and *Tn*25, indicating that *clntl*1, *Tn*21, *Tn*24 and *Tn*25 played significant roles in the propagation of the *sul*2 and *tet*G. Furthermore, the above four MGE genes also showed intense affinities with those potential hosts of *sul*2 and *tet*G. *Clntl*1 gene belongs to the integron gene, which can integrate exogenous genes such as ARGs as its own expression unit and transmit them on transposons or plasmids. *Tn*21, *Tn*24 and *Tn*25 genes belong to the transposase gene, which usually carries non-transposon genes such as ARGs for transposition, leading to the transmission of ARGs. Previous studies have suggested that tetracycline and sulphonamide resistance genes are coded on the same MGE genes to enable spread [32, 33]. Therefore, *sul*2 and *tet*G genes were probably coded on *clntl*1, *Tn*21, *Tn*24 or *Tn*25 genes of *Pseudomonas, Methylophilus*, *Methylobacillus*, *Trichoderma*, *Methanosarcina*, *Methanolobus* or *Methanomethylovorans* for propagation. The sum relative abundance of the *clntl*1, *Tn*21, *Tn*24 and *Tn*25 genes was in the order: closed-circuit group < open-circuit group < non-electrode group (Additional file 1: Figure S8). It suggests that not only could the soil MFC system inhibit or delay the occurrence of ARGs, but their potential propagation risk is also controlled. In the non-electrode treatment, antibiotic selective pressure causes microbial mutations or gene expression changes, resulting in amplification and transmission of ARGs. In the closed-circuit treatment, microbial activity is increased by biocurrent stimulation (self-induced circuits are formed in the presence of electrodes in the open-circuit group), which reduces microbial sensitivity to antibiotics, namely the selective pressure imposed by antibiotics rarely induces microbial genes mutations or expression changes in soil MFCs [34]. Besides, lower antibiotic concentration in soil MFCs may also lower its selective pressure on micro-organisms, thus reducing the probability of ARGs amplification and transmission.

**Fig. 7:**
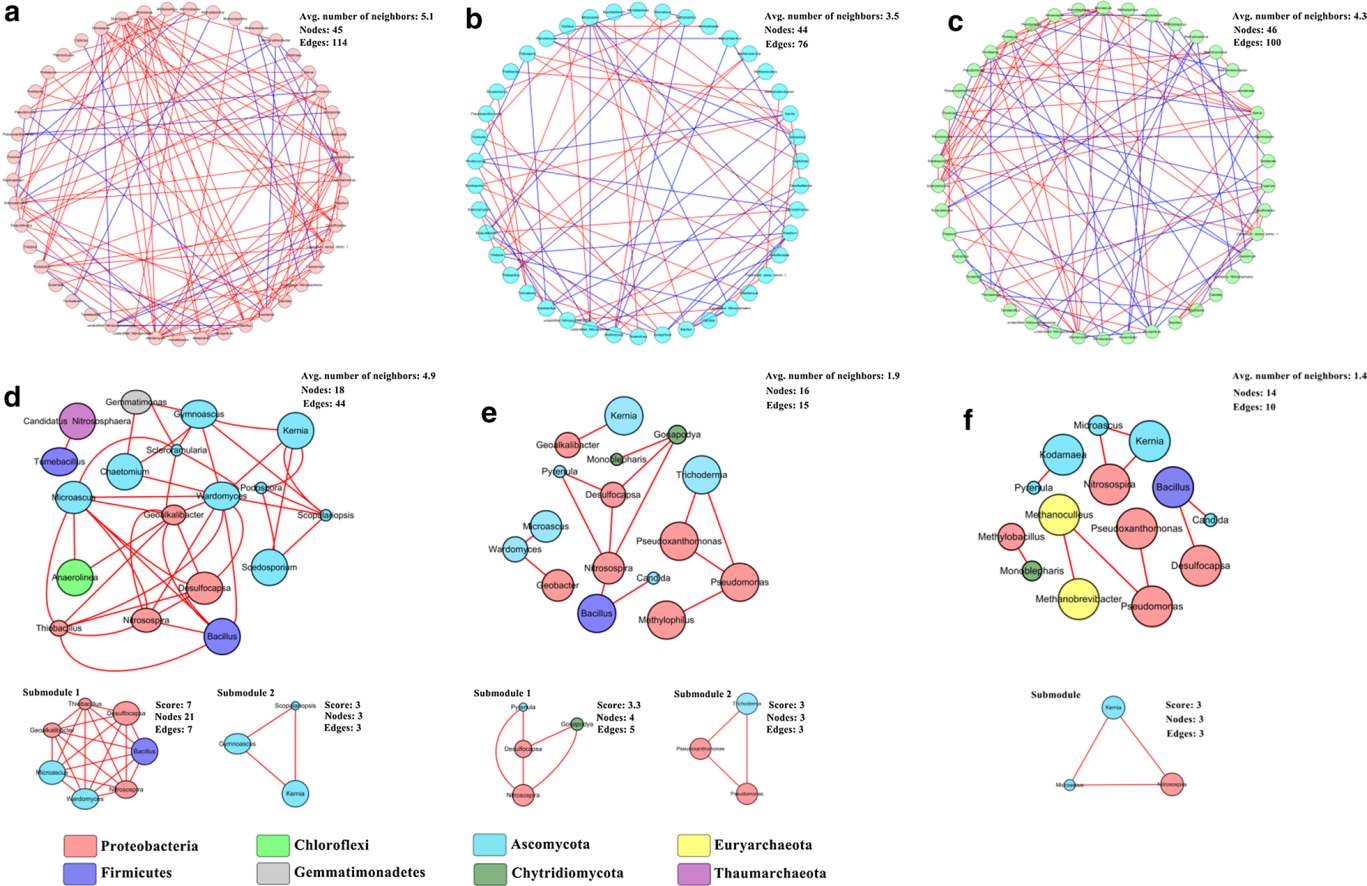
Networks of the closed-circuit group (**a**), open-circuit group (**b**) and non-electrode group (**c**) spiked with antibiotics are based on the correlation analysis of bacteria, fungi and archaea at the genus level. Red edges represent significant positive relationships, and blue edges represent significant negative relationships (*p* < 0.05, Spearman test). The microbes in closed-circuit (**d**), open-circuit (**e**) and non-electrode groups (**f**) spiked with antibiotics are further filtered by excluding the ones with the lower abundance than the corresponding non-antibiotic control. The size of each node is proportional to the abundance of the corresponding micro-organism

Compared with non-electrode and open-circuit treatments, the microbial links increased by 14–50% in closed-circuit treatments, in which the positive links accounted for 80% of the total links (Fig. 7a–c). By comparison, in open-circuit and non-electrode treatments, the positive links just, respectively, accounted for 57% and 59% of the total links. To explore interspecific relationships of keystones (functional microbes), the negative links between microbes were removed, and then, microbes were further filtered by excluding the ones with the lower abundance than the corresponding non-antibiotic control (Fig. 7d–f). Although the node number of new networks in different treatments was similar, the microbial affinities (edges) in the closed-circuit group were, respectively, 193% and 340% higher than those in the open-circuit group and non-electrode group. According to the network topology parameters, the new network in the closed-circuit group exhibited the optimal performance, followed by that in the open-circuit group (Additional file 1: Table S5). For instance, compared with the non-electrode group, the shortest paths in the open-circuit group increased by 121%, which further rose by 290% in the closed-circuit group. The shortest path represents the overall ability of the micro-organisms to influence their reciprocal activity or abundance for individual nodes (the higher the value is, the more stable the network is) [35]. It means that the microbial networks in the closed-circuit group and open-circuit group are more robust than that in the non-electrode group. Based on the above analysis, although the total microbial interspecific relationship in the open-circuit group was weaker than that in the non-electrode group (Fig. 7b, c), the affinities of keystones were higher than those in the non-electrode group (Fig. 7e, f). It suggests that some ineffective interspecific relationships for the biodegradation of antibiotics are discarded in the open-circuit group, which enhances the efficiency of biodegradation.

Not only had the affinities of functional microbes been strengthened by the introduction of electrodes and the stimulation of biocurrent, but also the microbial community structure had been changed. The submodules are performed to identify some closely related microbial groups in a network and to better reveal the interspecific relationships of micro-organisms [36, 37]. Two submodules were found in the closed-circuit group by the MCODE, in which the first submodule achieved a high network score of 7 (the closest links among the microbes of a submodule) (Fig. 7d). An extremely close links was observed among *Bacillus*, *Nitrosospira*, *Desulfocapsa*, *Geoalkalibacter*, *Thiobacillus*, *Microascus* and *Wardomyces*. In those microbes, *Bacillus* sp., *Desulfocapsa* sp. and *Geoalkalibacter* sp. are recognized as electrogenic micro-organisms [38–40]. *Nitrosospira* sp. and *Bacillus* sp. involved in nitrogen cycle [41]. Moreover, a previous study indicates that *Thiobacillus* sp. has a potential capacity to degrade phenol [42]. In terms of the above-mentioned evidences, a complex metabolic network involving the functions of electricity generation, degradation and nitrogen transformation was established in the soil MFC system. A previous study indicated that MFCs may decrease antibiotic pressure more effectively by selecting a stand-alone functional microbial taxon more rapidly, thereby reducing the abundance of ARGs [20]. Meanwhile, there were also two submodules in the open-circuit group; except for *Nitrosospira* and *Desulfocapsa*, the genera of *Pseudomonas*, *Pseudoxanthomonas* and *Trichoderma* have been reported to be degraders of benzene, toluene, ethylbenzene and xylene (BTEX) [43], DDT [44] and alachlor [45], respectively (Fig. 7e). By comparison, there was only one submodule in the non-electrode group (Fig. 7f).

## Conclusions

Soil MFCs possess advantages for the elimination of antibiotics and ARGs with sevenfold to eightfold higher electricity generation than that of the control treatment. Compared with sulphonamides, the enhancement removal of tetracycline is higher, while both potential ARG propagation risk is reduced in soil MFCs. The community structure and interspecific relationship of functional microbes had been reconstructed and strengthened in open- and closed-circuit treatments to adapt a disposed function of biodegradation. Some ineffective interspecific relationships for biodegradation of antibiotics were weakened after the introduction of electrodes. An intensive microbial community network capable of electricity generation, degradation and nitrogen transformation is established in closed-circuit treatments. Revealing these biological interactions is key for improving the versatile functions of bioelectrochemical remediation systems.

## Methods

### Tested soil and chemicals

Soils (0–20 cm in depth) were collected from farmland in Xiqing, Tianjin of China. The samples were air-dried, grinded and passed through a 2-mm sieve. No antibiotics were detected in this soil. The physicochemical properties of the soil are summarized in Additional file 1: Table S6. Tetracycline and sulfadiazine were purchased from Dr. Ehrenstorfer (Germany). Chemicals such as methanol, acetonitrile and acetone were of HPLC grade.

### Soil MFC configuration and operation

**Fig. 8:**
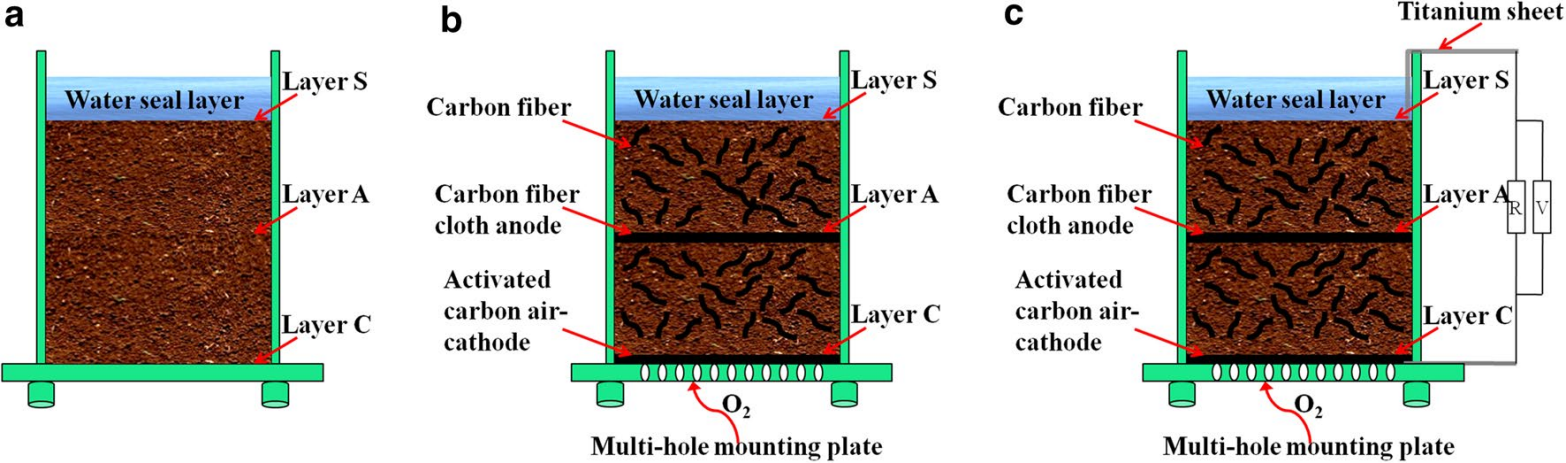
The soil MFCs construction of non-electrode group (**a**), open-circuit group (**b**) and closed-circuit group (**c**)

A single-chamber soil MFC (6 cm × 6 cm × 9 cm) was assembled with a carbon fibre cloth anode and an activated carbon air cathode (Fig. 8). The carbon fibre cloth 6 cm × 6 cm in size was cleaned overnight with acetone before use, and the activated carbon air cathode (6 cm × 6 cm) was manufactured using our previously described rolling-press method [46, 47]. An external resistance of 100 Ω was connected to the closed-circuit soil MFCs. Carbon fibres (1 cm in length) pretreated with acetone were mixed into the tested soil as 1% of the mass fraction to reduce soil internal resistance and enhance bioelectricity generation [17]. Each closed-circuit soil MFC had a duplicate as well as corresponding open and non-electrode controls. In addition, a non-electrode control had no anode, no cathode and no carbon fibres. All soil MFCs were filled with 300 g of the tested soil (with 120 mL of deionized water).

The experiment was divided into three groups. The first group contained closed-circuit soil MFCs (labelled as TC), an open-circuit MFC (TO) and a non-electrode control MFC (TN) spiked with tetracycline (5 mg kg^−1^). The second group included closed-circuit soil MFCs (SC), an open-circuit MFC (SO) and a non-electrode control MFC (SN) spiked with sulfadiazine (5 mg kg^−1^). The last group were the controls without the addition of antibiotics (respectively, marked as CC, CO and CN). Experimental design details are provided in Additional file 1: Table S7. All treatments were incubated at a constant temperature of 30 °C.

### Electrochemical and chemical analysis

The voltage (*U*) across the 100 Ω of external resistance (*R*) was recorded using a data acquisition system (PISO-813, ICP DAS Co., Ltd, Shanghai of China). Polarization and power density curves were monitored by modifying external resistances from 10,000 to 100 Ω. Samples were collected near the cathode (layer C), anode (layer A) and surface layer (layer S) in the soil MFCs (Fig. 8). Methods for determining the concentrations of tetracycline and sulfadiazine were modified from published protocols [48, 49]. Detailed methods are provided in Additional file 1.

### Target gene analysis

Soil samples were acquired from layers C, A and S for target gene and biological analyses. Genomic DNA extraction was based on a previously published method [50]. ARGs, 16S rRNA genes, integron genes and transposase genes were detected using a Smartchip real-time PCR system (WaferGen Biosystems, USA) by Microanaly (Shanghai) Gene Technologies Co., Ltd; the primer sequences used to detect these genes are listed in Additional file 1: Table S1. The programs of PCR were based on a previous literature [51]; each PCR was performed in 100 nL, with the following procedure: 10 min at 95 °C, followed by 40 cycles of the following program were used for amplification: denaturation at 95 °C for 30 s, annealing at 60 °C for 30 s. The results were analysed using a qPCR automatic quantitative software, which advances the credibility of the data by excluding the wells with multiple melting peaks or amplification efficiency beyond the range of 90–110%. Then, the target gene was considered to be detected when the threshold cycle (*C*_T_) value was less than 31 and the deviation of three duplications was lower than 20%.

### Biological analysis

Total genomic DNA in the soil samples was extracted using the CTAB method. DNA concentration and purity were detected in 1% agarose gels, and DNA was diluted to 1 ng μL^−1^ using sterile water.

Bacterial 16S rRNA gene fragments from the V4 region were amplified by a PCR method using the universal primers 515F (GTGCCAGCMGCCGCGGTAA) and 806R (GGACTACHVGGGTWTCTAAT). Fungal 16S rRNA gene fragments from the ITS1 region were amplified by PCR using the primers ITS5-1737F (GGAAGTAAAAGTCGTAACAAGG) and ITS2-2043R (GCTGCGTTCTTCATCG-ATGC). An archaeal 16S rRNA gene clone library was amplified by PCR using the primers 1106F (TTWAGTCAGGCAACGAGC) and 1378R (TGTGCAAGGAGCAG-GGAC). All PCRs were conducted with Phusion^®^ High-Fidelity PCR Master Mix (New England Biolabs). The same quantity of 1× loading buffer (containing SYB green) was mixed with the PCR products according to their concentrations, and the samples were subjected to electrophoresis on 2% agarose gels for detection. Samples with a bright main band around 300 bp for bacteria, 200–400 bp for fungi and 350–450 bp for archaea were selected for further examination. The target strips were purified using a Gel Extraction Kit (Qiagen). Sequencing libraries were built using a TruSeq^®^ DNA PCR-Free Sample Preparation Kit (Illumina, USA). The qualified libraries were assessed using a Qubit@ 2.0 Fluorometer (Thermo Scientific) as well as PCR quantitative detection and were sequenced using a HiSeq 2500 PE250 platform by the Novogene Company (Beijing, China).

### Network analysis

Networks were employed to reveal the relationships among target genes (ARGs, integron genes and transposase genes) and microbial abundances at the genus level (bacteria, fungi and archaea) using the Cytoscape 3.7.0 software. In order to reduce the network complexity, the possible Spearman’s rank correlations within and between target genes and microbial abundances were calculated, and a correlation between two items was considered statistically robust if *p* < 0.05 (Spearman test). Nodes represent target genes or microbes, edges represent the interaction between nodes, and neighbours are nodes connected by an edge [35]. To further understand the differences between networks, a series of network topology parameters (e.g. clustering coefficient, average number of neighbours, network density and shortest paths) were calculated by network analysis tools. Besides, modular structures and groups of highly interconnected nodes were analysed using the MCODE application with the standard parameters [36].

### Calculations

The current density (mA m^−2^) and power density (mW m^−2^) of each soil MFC were normalized to the project area of the air cathode (*A *= 0.0036 m^2^) and were calculated as *I*′ = *U*/(*R*·*A*) and *P*′ = *U*^2^/(*R*·*A*), respectively. The accumulated charge output was calculated as $Q = \int \nolimits_{0}^{T} \frac{U}{R}{\text{dt,}}$ and the cycle time (*T*) was 1800 s. The antibiotic removal efficiency (*β*) was calculated as *β* = (*C*′ − *C*)/*C*′ × 100%, where *C*′ was the concentration of the original soil and *C* was the antibiotic residue at the end of the experiment. The relative copy number of each target gene (*γ*) was calculated as *γ* = 10^(31−CT)/(10/3)^ [51]. One-way ANOVA (SPSS 18.0) was used to determine significant differences (*p* < 0.05, Duncan’s test) between samples, indicated by different lowercase letters. Bivariate (SPSS 18.0) was used to determine significant correlations (*p* < 0.05, Spearman test) between samples.

### Highlights

- Removal of antibiotics increased by 10–35% with 940–1132 C of charge output.
- Abundances of ARGs and propagation risk were decreased in soil MFCs.
- Open-circuit treatments showed a similar efficiency as closed-circuit MFCs.
- Ineffective microbial interactions reduced after the introduction of electrodes.
- Relations among key microbes were enhanced by the biocurrent stimulation.

## Additional file

**Additional file 1: Table S1.** List of target genes. **Table S2.** HiSeq sequencing data of bacterial, fungal and archaea communities. **Table S3.** The Alpha index of bacterial, fungal and archaea communities. **Table S4.** The Alpha index of bacterial, fungal and archaea communities in layers. **Table S5.** The network topology parameters of different treatments. **Table S6.** The physicochemical properties of experimental soil. **Table S7.** The experimental design. **Figure S1.** Change of voltage within the first 24 hour (a) and open-circuit voltage (b) of soil MFCs. Closed-circuit treatments spiked with tetracycline, sulfadiazine or without antibiotics added are marked as TC, SC or CC, respectively. An external resistance of 100 Ω was connected to each closed-circuit soil MFC. **Figure S2.** Detection rates of ARGs (*tet* and *sul* genes) and MGE genes in tested soils (*n* = 27). **Figure S3.** Taxonomic classification of bacterial DNA sequences from soil communities in different layers of MFCs at the phylum level (a), the class level distribution of the dominant phyla of *Proteobacteria* (b), *Firmicutes* (c), *Bacteroides* (d). CC/CO/CN/TC/TO/TN/SC/SO/SNC, CC/CO/CN/TC/TO/TN/SC/SO/SNA and CC/CO/CN/TC/TO/TN/SC/SO/SNS represent layer C, layer A and layer S in the same rector, respectively. **Figure S4.** Taxonomic classification of the microbial DNA sequences from soil communities in the MFCs at the genus level for bacteria (a), fungi (b) and archaea (c). **Figure S5.** Taxonomic classification of fungal DNA sequences from soil communities in different layers of MFCs the class level distribution of the dominant phyla of *Ascomycota* (a), *Zygomycota* (b), *Basidiomycota* (c), *Chytridiomycota* (d). **Figure S6.** Taxonomic classification of archaeal DNA sequences from soil communities in different layers of MFCs the class level distribution of the dominant phyla of *Euryarchaeota* (a), *Thaumarchaeota* (b). **Figure S7.** Comparison of current densities (a) and charge output (b) of soil MFCs between CC1 (in our study) and CC2. Cathodic microbial community adaptation to the removal of chlorinated herbicide in soil microbial fuel cells, Environmental Science and Pollution Research, 2018, 25(17), 16900-16912. **Figure S8.** The sum relative abundance of *Tn*21, *Tn*24, *Tn*25 and *cIntI*1 in treatments spiked with tetracycline and sulfadiazine.

## Data Availability

All data generated or analysed during this study are included in this published article and its additional information files.

## Abbreviations

ARGs: antibiotic resistance genes
MFCs: microbial fuel cells
MGE: mobile genetic element
TC: closed-circuit treatments spiked with tetracycline
SC: closed-circuit treatments spiked with sulfadiazine
CC: closed-circuit treatments without the addition of antibiotics
TO: open-circuit treatments spiked with tetracycline
SO: open-circuit treatments spiked with sulfadiazine
CO: open-circuit treatments without the addition of antibiotics
TN: non-electrode treatments spiked with tetracycline
SN: non-electrode treatments spiked with sulfadiazine
CN: non-electrode treatments without the addition of antibiotics
OCV: open-circuit voltages
OTUs: operational taxonomic units

## References

[1] Zhang QQ, Ying GG, Pan CG, Liu YS, Zhao JL (2015). Comprehensive evaluation of antibiotics emission and fate in the river basins of China: source analysis, multimedia modeling, and linkage to bacterial resistance. Environ Sci Technol.

[2] Wu XF, Wei YS, Zheng JX, Zhao X, Zhong WK (2011). The behavior of tetracyclines and their degradation products during swine manure composting. Bioresour Technol.

[3] Wang C, Qu GZ, Wang TC, Deng F, Liang DL (2018). Removal of tetracycline antibiotics from wastewater by pulsed corona discharge plasma coupled with natural soil particles. Chem Eng J.

[4] Wang FH, Qiao M, Chen Z, Su JQ, Zhu YG (2015). Antibiotic resistance genes in manure-amended soil and vegetables at harvest. J Hazard Mater.

[5] Ji XL, Shen QH, Liu F, Ma J, Xu G, Wang YL, Wu MH (2012). Antibiotic resistance gene abundances associated with antibiotics and heavy metals in animal manures and agricultural soils adjacent to feedlots in Shanghai, China. J Hazard Mater.

[6] Zhu YG, Johnson TA, Su JQ, Qiao M, Guo GX, Stedtfeld RD, Hashsham SA, Tiedje JM (2013). Diverse and abundant antibiotic resistance genes in Chinese swine farms. Proc Natl Acad Sci US A.

[7] Qiao M, Ying GG, Singer AC, Zhu YG (2018). Review of antibiotic resistance in China and its environment. Environ Int.

[8] Li XJ, Wang X, Weng LP, Zhou QX, Li YT (2017). Microbial fuel cells for organic-contaminated soil remedial applications: a review. Energy Technol.

[9] Lovley DR (2006). Bug juice: harvesting electricity with microorganisms. Nat Rev Microbiol.

[10] Li XJ, Wang X, Wan LL, Zhang YY, Li N, Li DS, Zhou QX (2016). Enhanced biodegradation of aged petroleum hydrocarbons in soils by glucose addition in microbial fuel cells. J Chem Technol Biotechnol.

[11] Yu B, Tian J, Feng L (2017). Remediation of PAH polluted soils using a soil microbial fuel cell: influence of electrode interval and role of microbial community. J Hazard Mater.

[12] Wang X, Cai Z, Zhou QX, Zhang ZN, Chen CH (2012). Bioelectrochemical stimulation of petroleum hydrocarbon degradation in saline soil using U-tube microbial fuel cells. Biotechnol Bioeng.

[13] Li XJ, Wang X, Zhang YY, Zhao Q, Yu BB, Li YT, Zhou QX (2016). Salinity and conductivity amendment of soil enhanced the bioelectrochemical degradation of petroleum hydrocarbons. Sci Rep.

[14] Huang DY, Zhou SG, Chen Q, Zhao B, Yuan Y, Zhuang L (2011). Enhanced anaerobic degradation of organic pollutants in a soil microbial fuel cell. Chem Eng J.

[15] Cao X, Song HL, Yu CY, Li XN (2015). Simultaneous degradation of toxic refractory organic pesticide and bioelectricity generation using a soil microbial fuel cell. Bioresour Technol.

[16] Li XJ, Wang X, Ren ZJ, Zhang YY, Li N, Zhou QX (2015). Sand amendment enhances bioelectrochemical remediation of petroleum hydrocarbon contaminated soil. Chemosphere.

[17] Li XJ, Wang X, Zhao Q, Wan LL, Li YT, Zhou QX (2016). Carbon fiber enhanced bioelectricity generation in soil microbial fuel cells. Biosens Bioelectron.

[18] Wang L, Liu YL, Ma J, Zhao F (2016). Rapid degradation of sulphamethoxazole and the further transformation of 3-amino-5-methylisoxazole in a microbial fuel cell. Water Res.

[19] Song HL, Li H, Zhang S, Yang YL, Zhang LM, Xu H, Yang XL (2018). Fate of sulfadiazine and its corresponding resistance genes in up-flow microbial fuel cell coupled constructed wetlands: effects of circuit operation mode and hydraulic retention time. Chem Eng J.

[20] Yan WF, Guo YY, Xiao Y, Wang SH, Ding R, Jiang JQ, Gang HY, Wang H, Yang J, Zhao F (2018). The changes of bacterial communities and antibiotic resistance genes in microbial fuel cells during long-term oxytetracycline processing. Water Res.

[21] Catal T, Yavaser S, Enisoglu-Atalay V, Bermek H, Ozilhan S (2018). Monitoring of neomycin sulfate antibiotic in microbial fuel cells. Bioresour Technol.

[22] Wang L, You LX, Zhang JM, Yang T, Zhang W, Zhang ZX, Liu PX, Wu S, Zhao F, Ma J (2018). Biodegradation of sulfadiazine in microbial fuel cells: reaction mechanism, biotoxicity removal and the correlation with reactor microbes. J Hazard Mater.

[23] Lu L, Xing DF, Ren ZY (2015). Microbial community structure accompanied with electricity production in a constructed wetland plant microbial fuel cell. Bioresour Technol.

[24] Hari AR, Katuri KP, Logan BE, Saikaly PE (2016). Set anode potentials affect the electron fluxes and microbial community structure in propionate-fed microbial electrolysis cells. Sci Rep.

[25] Lu L, Ren ZY (2016). Microbial electrolysis cells for waste biorefinery: a state of the art review. Bioresour Technol.

[26] Li Y, Li XJ, Sun Y, Zhao XD, Li YT (2018). Cathodic microbial community adaptation to the removal of chlorinated herbicide in soil microbial fuel cells. Environ Sci Pollut Res.

[27] Wang Y, Zhang H, Zhang JH, Lu C, Huang QQ, Wu J, Liu F (2011). Degradation of tetracycline in aqueous media by ozonation in an internal loop-lift reactor. J Hazard Mater.

[28] Zhang S, Song HL, Yang XL, Yang KY, Wang XY (2016). Effect of electrical stimulation on the fate of sulfamethoxazole and tetracycline with their corresponding resistance genes in three-dimensional biofilm-electrode reactors. Chemosphere.

[29] Wang J, He MF, Zhang DL, Ren ZY, Song TS, Xie JJ (2017). Simultaneous degradation of tetracycline by a microbial fuel cell and its toxicity evaluation by zebrafish. RSC Adv.

[30] Pan SH, Yan N, Liu XY, Wang WB, Zhang YM, Liu R, Rittmann BE (2014). How UV photolysis accelerates the biodegradation and mineralization of sulfadiazine (SD). Biodegradation.

[31] Park JH, Kang HJ, Park KH, Park HD (2018). Direct interspecies electron transfer via conductive materials: a perspective for anaerobic digestion applications. Bioresour Technol.

[32] Nonaka L, Maruyama F, Miyamoto M, Miyakoshi M, Kurokawa K, Masuda M (2012). Novel conjugative transferable multiple drug resistance plasmid pAQU1 from *Photobacterium damselae* subsp. *damselae* isolated from marine aquaculture environment. Microbes Environ.

[33] Jang HM, Kim YB, Choi S, Lee Y, Shin SG, Unno T, Kim YM (2018). Prevalence of antibiotic resistance genes from effluent of coastal aquaculture, South Korea. Environ Pollut.

[34] Yan WF, Xiao Y, Yan WD, Ding R, Wang SH, Zhao F (2019). The effect of bioelectrochemical systems on antibiotics removal and antibiotic resistance genes: a review. Chem Eng J.

[35] Rodríguez-Valdecantos G, Manzano M, Sánchez R, Urbina F, Hengst MB, Lardies MA, Ruz GA, González B (2017). Early successional patterns of bacterial communities in soil microcosms reveal changes in bacterial community composition and network architecture, depending on the successional condition. Appl Soil Ecol.

[36] Zheng W, Zhao ZY, Gong QL, Zhai BN, Li ZY (2018). Responses of fungal–bacterial community and network to organic inputs vary among different spatial habitats in soil. Soil Biol Biochem.

[37] Halary S, Leigh JW, Cheaib B, Lopez P, Bapteste E (2010). Network analyses structure genetic diversity in independent genetic worlds. Proc Natl Acad Sci USA.

[38] Pierra M, Carmona-Martínez AA, Trably E, Godon JJ, Bernet N (2015). Specific and efficient electrochemical selection of *Geoalkalibacter subterraneus* and *Desulfuromonas acetoxidans* in high current-producing biofilms. Bioelectrochemistry.

[39] You LX, Liu LD, Xiao Y, Dai YF, Chen BL, Jiang YX, Zhao F (2018). Flavins mediate extracellular electron transfer in Gram-positive *Bacillus megaterium* strain LLD-1. Bioelectrochemistry.

[40] Stams AJM, de Bok FAM, Plugge CM, van Eekert MHA, Dolfing J, Schraa G (2006). Exocellular electron transfer in anaerobic microbial communities. Environ Microbiol.

[41] Fang WS, Yan DD, Wang QX, Huang B, Ren ZJ, Wang XL, Wang XN, Li Y, Ouyang CB, Migheli Q, Cao AC (2019). Changes in the abundance and community composition of different nitrogen cycling groups in response to fumigation with 1,3-dichloropropene. Sci Total Environ.

[42] Oshiki M, Masuda Y, Yamaguchi T, Araki N (2018). Synergistic inhibition of anaerobic ammonium oxidation (anammox) activity by phenol and thiocyanate. Chemosphere.

[43] Hassan HA, Aly AA (2018). Isolation and characterization of three novel catechol 2,3-dioxygenase from three novel haloalkaliphilic BTEX-degrading *Pseudomonas* strains. Int J Biol Macromol.

[44] Wang GL, Bi M, Liang B, Jiang JD, Li SP (2011). *Pseudoxanthomonas jiangsuensis* sp nov., a DDT-degrading bacterium isolated from a long-term DDT-polluted soil. Curr Microbiol.

[45] Nykiel-Szymańska J, Bernat P, Słaba M (2018). Potential of *Trichoderma koningii* to eliminate alachlor in the presence of copper ions. Ecotoxicol Environ Saf.

[46] Li XJ, Wang X, Zhang YY, Ding N, Zhou QX (2014). Opening size optimization of metal matrix in rolling-pressed activated carbon air–cathode for microbial fuel cells. Appl Energy.

[47] Li XJ, Wang X, Zhang YY, Cheng LJ, Liu J, Li F, Gao BL, Zhou QX (2014). Extended petroleum hydrocarbon bioremediation in saline soil using Pt-free multianodes microbial fuel cells. RSC Adv.

[48] Martínez-Carballo E, González-Barreiro C, Scharf S, Gans O (2007). Environmental monitoring study of selected veterinary antibiotics in animal manure and soils in Austria. Environ Pollut.

[49] Hu W, Ma LL, Guo CS, Sha J, Zhu XW, Wang YQ (2012). Simultaneous extraction and determination of fluoroquinolones, tetracyclines and sulfonamides antibiotics in soils using optimised solid phase extraction chromatography-tandem mass spectrometry. Int J Environ Anal Chem.

[50] Yu BB, Wang X, Yu S, Li Q, Zhou QX (2014). Effects of roxithromycin on ammonia-oxidizing bacteria and nitrite-oxidizing bacteria in the rhizosphere of wheat. Appl Microbiol Biotechnol.

[51] Chen QL, An XL, Li H, Su JQ, Ma YB, Zhu YG (2016). Long-term field application of sewage sludge increases the abundance of antibiotic resistance genes in soil. Environ Int.

