# Supplementary material for: Shifting interactions among bacteria, fungi and archaea enhance removal of antibiotics and antibiotic resistance genes in the soil bioelectrochemical remediation
Source: Biotechnol Biofuels. 2019 Jun 24;12:160. doi: 10.1186/s13068-019-1500-1 (PMC6589883; doi:10.1186/s13068-019-1500-1)
Supplement: Supplementary file 1 — Additional file 1: Table S1. List of target genes. Table S2. HiSeq sequencing data of bacterial, fungal and archaea communities. Table S3. The Alpha index of bacterial, fungal and archaea communities. Table S4. The Alpha index of bacterial, fungal and archaea communities in layers. Table S5. The network topology parameters of different treatments. Table S6. The physicochemical properties of experimental soil. Table S7. The experimental design. Figure S1. Change of voltage within the first 24 hour (a) and open-circuit voltage (b) of soil MFCs. Closed-circuit treatments spiked with tetracycline, sulfadiazine or without antibiotics added are marked as TC, SC or CC, respectively. An external resistance of 100 Ω was connected to each closed-circuit soil MFC. Figure S2. Detection rates of ARGs (tet and sul genes) and MGE genes in tested soils (n = 27). Figure S3. Taxonomic classification of bacterial DNA sequences from soil communities in different layers of MFCs at the phylum level (a), the class level distribution of the dominant phyla of Proteobacteria (b), Firmicutes (c), Bacteroides (d). CC/CO/CN/TC/TO/TN/SC/SO/SNC, CC/CO/CN/TC/TO/TN/SC/SO/SNA and CC/CO/CN/TC/TO/TN/SC/SO/SNS represent layer C, layer A and layer S in the same rector, respectively. Figure S4. Taxonomic classification of the microbial DNA sequences from soil communities in the MFCs at the genus level for bacteria (a), fungi (b) and archaea (c). Figure S5. Taxonomic classification of fungal DNA sequences from soil communities in different layers of MFCs the class level distribution of the dominant phyla of Ascomycota (a), Zygomycota (b), Basidiomycota (c), Chytridiomycota (d). Figure S6. Taxonomic classification of archaeal DNA sequences from soil communities in different layers of MFCs the class level distribution of the dominant phyla of Euryarchaeota (a), Thaumarchaeota (b). Figure S7. Comparison of current densities (a) and charge output (b) of soil MFCs between CC1 (in our study) and CC2. Cathodic m [file 13068_2019_1500_MOESM1_ESM.doc]

***--Additional file—***

**Shifting interactions among bacteria, fungi and archaea enhance removal of antibiotics and antibiotic resistance genes in the soil bioelectrochemical remediation**

Xiaodong Zhao ^1^, Xiaojing Li ^1^,*, Yue Li ^1^, Yang Sun ^1^, Xiaolin Zhang ^1^, Liping Weng ^1^, Tianzhi Ren ^1^, Yongtao Li ^1, 2^,*

^1^ Agro-Environmental Protection Institute, Ministry of Agriculture and Rural Affairs / Key Laboratory of Original Agro-Environmental Pollution Prevention and Control, MARA / Tianjin Key Laboratory of Agro-Environment and Agro-Product Safety, Tianjin 300191, China

^2^ College of Natural Resources and Environment, South China Agricultural University, Guangzhou 510642, China

* Corresponding Authors: Phone: (86)22-23611021; Fax: (86)22-23613820;

E-mail: lixiaojing@caas.cn (Li X.), yongtao@scau.edu.cn (Li Y.)

Page: 17

Number of Table: 7

Number of Figure: 8

**Methods for the determination of tetracycline and sulfadiazine:**

1 g of the tested soil was mixed with 15 mL of Na_2_EDTA-McIlvaine buffer (pH 4.2) with methanol (1:1, *v*:*v*). After vortexing (2500 rpm, 10 min), ultrasonication (100 kHz, 30 min) and centrifugation (10000 g, 10 min), the supernatant was transferred into a 50-mL centrifuge tube. The operation was repeated twice. The supernatants were blended and concentrated by a rotary evaporator to a constant volume and then diluted to 100 mL with ultrapure water. After filtering through a 0.45-μm nylon filter, the extract was cleaned using Oasis HLB (6 cm^3^, 500 mg) cartridges (Waters, U.S.). The eluent was blown to near dryness with mild nitrogen at 50 °C, and then re-dissolved using 1 mL mixed solution of 20% methanol and 80% phosphoric acid (0.1%). After filtering through a 0.22-μm nylon filter, the extract was measured by a UPLC system with a Photo-Diode Array detector (Waters, U.S.). The ACQUITY UPLC® HSS T3 column (2.1 mm×100 mm, 1.8 μm) was used at 40 °C, and the detection wavelength was set at 274 nm. The mobile phases were 0.1% phosphoric acid (A) and acetonitrile (B), the flow rate was 0.4 mL⋅min^-1^, and the injection volume was 10 μL. The gradient elution programme was as follows: 0-1 min, 10% B; 1-5 min, 10-80% B; 5-6 min, 80% B; 6-7 min, 80-10% B; 7-9 min, 10% B.

**Table S1 List of target genes.**

| **Gene Name** | **Forward Primer** | **Reverse Primer** | **class** |
| --- | --- | --- | --- |
| 16S rRNA | GGGTTGCGCTCGTTGC | ATGGYTGTCGTCAGCTCGTG |  |
| *cIntI*-1(class1) | GGCATCCAAGCAGCAAG | AAGCAGACTTGACCTGA | Integron |
| *intI*-1(clinic) | CGAACGAGTGGCGGAGGGTG | TACCCGAGAGCTTGGCACCCA | Integron |
| *IS*613 | AGGTTCGGACTCAATGCAACA | TTCAGCACATACCGCCTTGAT | Transposase |
| *sul*1 | CAGCGCTATGCGCTCAAG | ATCCCGCTGCGCTGAGT | Sulfonamide |
| *sul*2 | TCATCTGCCAAACTCGTCGTTA | GTCAAAGAACGCCGCAATGT | Sulfonamide |
| *sul*A/*fol*P-01 | CAGGCTCGTAAATTGATAGCAGAAG | CTTTCCTTGCGAATCGCTTT | Sulfonamide |
| *sul*A/*fol*P-03 | CACGGCTTCGGCTCATGT | TGCCATCCTGTGACTAGCTACGT | Sulfonamide |
| *tet*(32) | CCATTACTTCGGACAACGGTAGA | CAATCTCTGTGAGGGCATTTAACA | Tetracycline |
| *tet*(34) | CTTAGCGCAAACAGCAATCAGT | CGGTGATACAGCGCGTAAACT | Tetracycline |
| *tet*(35) | ACCCCATGACGTACCTGTAGAGA | CAACCCACACTGGCTACCAGTT | Tetracycline |
| *tet*(36)-01 | AGAATACTCAGCAGAGGTCAGTTCCT | TGGTAGGTCGATAACCCGAAAAT | Tetracycline |
| *tet*(36)-02 | TGCAGGAAAGACCTCCATTACAG | CTTTGTCCACACTTCCACGTACTATG | Tetracycline |
| *tet*(37) | GAGAACGTTGAAAAGGTGGTGAA | AACCAAGCCTGGATCAGTCTCA | Tetracycline |
| *tet*A-01 | GCTGTTTGTTCTGCCGGAAA | GGTTAAGTTCCTTGAACGCAAACT | Tetracycline |
| *tet*A-02 | CTCACCAGCCTGACCTCGAT | CACGTTGTTATAGAAGCCGCATAG | Tetracycline |
| *tet*B-01 | AGTGCGCTTTGGATGCTGTA | AGCCCCAGTAGCTCCTGTGA | Tetracycline |
| *tet*B-02 | GCCCAGTGCTGTTGTTGTCAT | TGAAAGCAAACGGCCTAAATACA | Tetracycline |
| *tet*C-01 | CATATCGCAATACATGCGAAAAA | AAAGCCGCGGTAAATAGCAA | Tetracycline |
| *tet*C-02 | ACTGGTAAGGTAAACGCCATTGTC | ATGCATAAACCAGCCATTGAGTAAG | Tetracycline |
| *tet*D-01 | TGCCGCGTTTGATTACACA | CACCAGTGATCCCGGAGATAA | Tetracycline |
| *tet*D-02 | TGTCATCGCGCTGGTGATT | CATCCGCTTCCGGGAGAT | Tetracycline |
| *tet*E | TTGGCGCTGTATGCAATGAT | CGACGACCTATGCGATCTGA | Tetracycline |
| *tet*G-01 | TCAACCATTGCCGATTCGA | TGGCCCGGCAATCATG | Tetracycline |
| *tet*G-02 | CATCAGCGCCGGTCTTATG | CCCCATGTAGCCGAACCA | Tetracycline |
| *tet*H | TTTGGGTCATCTTACCAGCATTAA | TTGCGCATTATCATCGACAGA | Tetracycline |
| *tet*J | GGGTGCCGCATTAGATTACCT | TCGTCCAATGTAGAGCATCCATA | Tetracycline |
| *tet*K | CAGCAGTCATTGGAAAATTATCTGATTATA | CCTTGTACTAACCTACCAAAAATCAAAATA | Tetracycline |
| *tet*L-01 | AGCCCGATTTATTCAAGGAATTG | CAAATGCTTTCCCCCTGTTCT | Tetracycline |
| *tet*L-02 | ATGGTTGTAGTTGCGCGCTATAT | ATCGCTGGACCGACTCCTT | Tetracycline |
| *tet*M-01 | CATCATAGACACGCCAGGACATAT | CGCCATCTTTTGCAGAAATCA | Tetracycline |
| *tet*M-02 | TAATATTGGAGTTTTAGCTCATGTTGATG | CCTCTCTGACGTTCTAAAAGCGTATTAT | Tetracycline |
| *tet*O-01 | ATGTGGATACTACAACGCATGAGATT | TGCCTCCACATGATATTTTTCCT | Tetracycline |
| *tet*PA | AGTTGCAGATGTGTATAGTCGTAAACTATCTATT | TGCTACAAGTACGAAAACAAAACTAGAA | Tetracycline |
| *tet*Q | CGCCTCAGAAGTAAGTTCATACACTAAG | TCGTTCATGCGGATATTATCAGAAT | Tetracycline |
| *tet*R-02 | CGCGATAGACGCCTTCGA | TCCTGACAACGAGCCTCCTT | Tetracycline |
| *tet*R-03 | CGCGATGGAGCAAAAGTACAT | AGTGAAAAACCTTGTTGGCATAAAA | Tetracycline |
| *tet*S | TTAAGGACAAACTTTCTGACGACATC | TGTCTCCCATTGTTCTGGTTCA | Tetracycline |
| *tet*T | CCATATAGAGGTTCCACCAAATCC | TGACCCTATTGGTAGTGGTTCTATTG | Tetracycline |
| *tet*U-01 | GTGGCAAAGCAACGGATTG | TGCGGGCTTGCAAAACTATC | Tetracycline |
| *tet*V | GCGGGAACGACGATGTATATC | CCGCTATCTCACGACCATGAT | Tetracycline |
| *tet*X | AAATTTGTTACCGACACGGAAGTT | CATAGCTGAAAAAATCCAGGACAGTT | Tetracycline |
| *Tn*21 | CATCATCGGACGGACAGAATT | GTCGGAGATGTGGGTGTAGAAAGT | Transposase |
| *Tn*22 | GGGCGGGTCGATTGAAA | GTGGGCGGGATCTGCTT | Transposase |
| *Tn*23 | GAAACCGATGCTACAATATCCAATTT | CAGCACCGTTTGCAGTGTAAG | Transposase |
| *Tn*24 | GCCGCACTGTCGATTTTTATC | GCGGGATCTGCCACTTCTT | Transposase |
| *Tn*25 | CCGATCACGGAAAGCTCAAG | GGCTCGCATGACTTCGAATC | Transposase |
| *Tnp*A | AATTGATGCGGACGGCTTAA | TCACCAAACTGTTTATGGAGTCGTT | Transposase |

**Table S2** HiSeq sequencing data of bacterial, fungal and archaea communities.

| Sample name | Effective tags(#) | | | AvgLen(nt) | | | OTU numbers | | | Effective (%) | | | Goods coverage (%) | | |
| --- | --- | --- | --- | --- | --- | --- | --- | --- | --- | --- | --- | --- | --- | --- | --- |
|  | Bacteria | Fungi | Archaea | Bacteria | Fungi | Archaea | Bacteria | Fungi | Archaea | Bacteria | Fungi | Archaea | Bacteria | Fungi | Archaea |
| TCC | 80618 | 89657 | 90871 | 253 | 223 | 280 | 5077 | 757 | 403 | 95.04 | 96.65 | 96.36 | 0.985 | 0.997 | 0.999 |
| TCA | 90764 | 85194 | 75515 | 253 | 223 | 280 | 4954 | 723 | 335 | 94.94 | 97.02 | 96.40 | 0.985 | 0.997 | 0.998 |
| TCS | 84169 | 93433 | 81824 | 253 | 230 | 280 | 4373 | 651 | 338 | 93.76 | 96.84 | 95.45 | 0.986 | 0.997 | 0.999 |
| TOC | 93948 | 95683 | 87430 | 253 | 219 | 280 | 5079 | 678 | 260 | 96.33 | 96.40 | 96.95 | 0.984 | 0.996 | 0.999 |
| TOA | 94253 | 80791 | 77954 | 253 | 214 | 281 | 5138 | 736 | 127 | 94.91 | 88.64 | 97.16 | 0.984 | 0.996 | 1.000 |
| TOS | 76792 | 84906 | 93062 | 253 | 223 | 279 | 4421 | 750 | 1006 | 94.87 | 94.71 | 94.87 | 0.987 | 0.996 | 0.997 |
| TNC | 92340 | 75246 | 94890 | 253 | 221 | 280 | 5262 | 783 | 492 | 94.90 | 93.64 | 95.64 | 0.983 | 0.997 | 0.998 |
| TNA | 95166 | 94009 | 85472 | 253 | 219 | 280 | 4935 | 773 | 306 | 95.77 | 94.95 | 95.17 | 0.984 | 0.996 | 0.999 |
| TNS | 89735 | 73564 | 84624 | 253 | 228 | 281 | 4509 | 572 | 398 | 95.13 | 95.37 | 95.21 | 0.985 | 0.997 | 0.998 |
| SCC | 86437 | 83331 | 79804 | 253 | 220 | 279 | 4924 | 594 | 314 | 94.48 | 95.82 | 94.95 | 0.985 | 0.997 | 0.999 |
| SCA | 73327 | 89340 | 88242 | 253 | 222 | 280 | 4359 | 689 | 308 | 95.45 | 95.20 | 94.15 | 0.989 | 0.997 | 1.000 |
| SCS | 84932 | 91305 | 84416 | 253 | 220 | 278 | 1757 | 400 | 366 | 94.88 | 95.63 | 94.84 | 0.995 | 0.998 | 1.000 |
| SOC | 80996 | 90851 | 80485 | 253 | 219 | 280 | 4866 | 778 | 284 | 94.90 | 94.89 | 95.89 | 0.986 | 0.996 | 0.999 |
| SOA | 85400 | 90431 | 91048 | 253 | 218 | 280 | 4812 | 722 | 270 | 94.70 | 95.33 | 96.34 | 0.985 | 0.996 | 0.999 |
| SOS | 77740 | 82213 | 86143 | 253 | 224 | 279 | 4320 | 859 | 413 | 94.27 | 92.61 | 95.98 | 0.986 | 0.996 | 0.998 |
| SNC | 86234 | 93660 | 79640 | 253 | 220 | 280 | 5132 | 769 | 205 | 95.42 | 94.11 | 95.76 | 0.984 | 0.996 | 0.999 |
| SNA | 94432 | 88560 | 83456 | 253 | 219 | 280 | 5022 | 789 | 355 | 94.58 | 94.93 | 95.83 | 0.984 | 0.996 | 0.999 |
| SNS | 90509 | 86636 | 80395 | 253 | 216 | 279 | 3643 | 740 | 161 | 95.10 | 91.08 | 92.19 | 0.989 | 0.996 | 1.000 |
| CCC | 87304 | 82674 | 79998 | 253 | 222 | 280 | 5329 | 687 | 292 | 94.91 | 95.36 | 96.75 | 0.984 | 0.997 | 0.999 |
| CCA | 85238 | 85205 | 85094 | 253 | 220 | 280 | 4912 | 702 | 325 | 94.82 | 95.57 | 96.24 | 0.985 | 0.997 | 0.999 |
| CCS | 92793 | 87925 | 85009 | 253 | 221 | 283 | 4358 | 406 | 277 | 94.50 | 97.62 | 96.17 | 0.985 | 0.998 | 0.999 |
| COC | 88690 | 94641 | 90432 | 253 | 224 | 280 | 5392 | 717 | 239 | 95.53 | 95.53 | 93.77 | 0.983 | 0.996 | 0.999 |
| COA | 89641 | 93602 | 83739 | 253 | 220 | 280 | 4870 | 653 | 276 | 95.51 | 95.92 | 96.67 | 0.984 | 0.996 | 0.999 |
| COS | 87414 | 78241 | 91936 | 253 | 220 | 280 | 4564 | 646 | 430 | 95.02 | 95.79 | 96.40 | 0.985 | 0.997 | 0.998 |
| CNC | 78195 | 80706 | 78596 | 253 | 224 | 280 | 4734 | 656 | 276 | 94.56 | 95.32 | 93.34 | 0.986 | 0.997 | 0.999 |
| CNA | 83626 | 80329 | 79994 | 253 | 221 | 280 | 4697 | 648 | 406 | 95.75 | 95.55 | 96.45 | 0.985 | 0.997 | 0.999 |
| CNS | 89275 | 52654 | 81866 | 253 | 220 | 280 | 4482 | 562 | 213 | 95.10 | 95.66 | 95.34 | 0.984 | 0.999 | 0.999 |

**Table S3** The Alpha index of bacterial, fungal and archaea communities.

|  | Shannon | | | Chao1 | | |
| --- | --- | --- | --- | --- | --- | --- |
|  | Bacteria | Fungi | Archaea | Bacteria | Fungi | Archaea |
| TC | 9.58 | 4.52 | 4.47 | 4662 | 770 | 569 |
| TO | 9.65 | 4.21 | 4.68 | 4741 | 790 | 470 |
| TN | 9.26 | 4.12 | 4.69 | 4727 | 727 | 419 |
| SC | 8.46 | 3.87 | 4.17 | 3544 | 613 | 329 |
| SO | 9.50 | 4.13 | 4.18 | 4581 | 846 | 316 |
| SN | 9.48 | 4.59 | 4.13 | 4444 | 858 | 242 |
| CC | 9.29 | 3.65 | 4.33 | 4710 | 655 | 310 |
| CO | 9.58 | 4.08 | 4.36 | 4924 | 716 | 318 |
| CN | 9.30 | 4.25 | 4.47 | 4561 | 642 | 284 |

**Table S4** The Alpha index of bacterial, fungal and archaea communities in layers.

|  | Shannon | | | Chao1 | | |
| --- | --- | --- | --- | --- | --- | --- |
|  | Bacteria | Fungi | Archaea | Bacteria | Fungi | Archaea |
| TCC | 9.93 | 4.84 | 4.47 | 5012 | 783 | 376 |
| TCA | 9.76 | 4.55 | 4.41 | 4776 | 768 | 987 |
| TCS | 9.04 | 4.18 | 4.54 | 4197 | 758 | 344 |
| TOC | 9.97 | 4.23 | 4.28 | 4952 | 764 | 255 |
| TOA | 9.83 | 4.02 | 4.55 | 4964 | 790 | 134 |
| TOS | 9.16 | 4.38 | 5.21 | 4307 | 817 | 1020 |
| TNC | 9.82 | 4.53 | 4.78 | 5112 | 744 | 521 |
| TNA | 9.48 | 3.70 | 4.52 | 4769 | 869 | 299 |
| TNS | 8.49 | 4.13 | 4.75 | 4301 | 568 | 436 |
| SCC | 9.52 | 3.98 | 4.51 | 4779 | 664 | 312 |
| SCA | 9.44 | 3.81 | 4.11 | 4126 | 762 | 305 |
| SCS | 6.42 | 3.81 | 3.88 | 1726 | 413 | 370 |
| SOC | 9.94 | 3.78 | 4.00 | 4739 | 812 | 282 |
| SOA | 9.65 | 3.74 | 3.98 | 4739 | 842 | 268 |
| SOS | 8.90 | 4.87 | 4.57 | 4264 | 885 | 397 |
| SNC | 9.82 | 4.41 | 4.26 | 5018 | 848 | 216 |
| SNA | 9.76 | 3.98 | 4.45 | 4832 | 804 | 354 |
| SNS | 8.87 | 5.39 | 3.69 | 3482 | 921 | 158 |
| CCC | 10.07 | 4.22 | 4.33 | 5164 | 724 | 306 |
| CCA | 9.61 | 3.90 | 4.23 | 4760 | 801 | 348 |
| CCS | 8.19 | 2.83 | 4.44 | 4205 | 441 | 276 |
| COC | 10.02 | 4.34 | 4.56 | 5336 | 791 | 231 |
| COA | 9.47 | 3.82 | 4.18 | 4844 | 720 | 263 |
| COS | 9.25 | 4.06 | 4.34 | 4591 | 637 | 460 |
| CNC | 9.57 | 4.24 | 4.62 | 4601 | 721 | 258 |
| CNA | 9.52 | 3.95 | 4.43 | 4627 | 705 | 385 |
| CNS | 8.82 | 4.55 | 4.34 | 4454 | 499 | 210 |

**Table S5 The network topology parameters of different treatments.**

|  | Closed-circuit group | Open-circuit group | Non-electrode group |
| --- | --- | --- | --- |
| Clustering coefficient | 0.68 | 0.29 | 0.21 |
| Shortest paths | 242 (79%) | 62 (25%) | 28 (15%) |
| Average number of neighbors | 4.89 | 1.88 | 1.43 |
| Network density | 0.29 | 0.13 | 0.11 |

**Table S6** The physicochemical properties of experimental soil.

| Index | pH | Electrical  conductivity  (μS⋅cm^-1^) | Total  nitrogen  (g⋅kg^-1^) | Total  phosphorus  (g⋅kg^-1^) | Organic  matter  (g⋅kg^-1^) |
| --- | --- | --- | --- | --- | --- |
| Values | 7.72 | 517.20 | 1.40 | 0.94 | 23.97 |

**Table S7 The experimental design.**

| Treatments | Soil MFCs types | Mixing carbon fiber in soil (%) | Labels | Concentration (mg⋅kg^-1^) |
| --- | --- | --- | --- | --- |
| Treatments spiked with tetracycline | Closed-circuit | 1 | TC | 5 |
|  | Open-circuit | 1 | TO | 5 |
|  | Non-electrode | 0 | TN | 5 |
| Treatments spiked with sulfadiazine | Closed-circuit | 1 | SC | 5 |
|  | Open-circuit | 1 | SO | 5 |
|  | Non-electrode | 0 | SN | 5 |
| Non-antibiotic control treatments | Closed-circuit | 1 | CC | 0 |
|  | Open-circuit | 1 | CO | 0 |
|  | Non-electrode | 0 | CN | 0 |


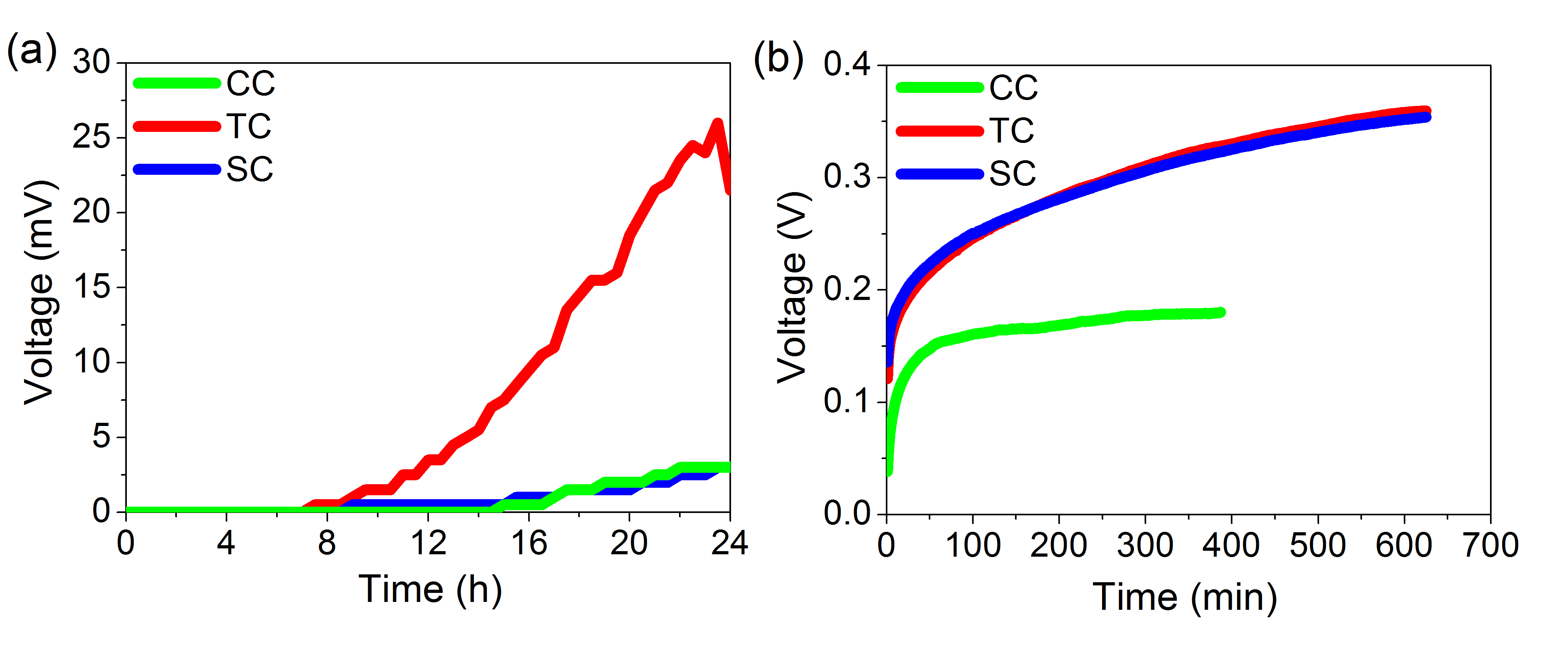


**Fig. S1.** Change of voltage within the first 24 hour (a) and open-circuit voltage (b) of soil MFCs. Closed-circuit treatments spiked with tetracycline, sulfadiazine or without antibiotics added are marked as TC, SC or CC, respectively. An external resistance of 100 Ω was connected to each closed-circuit soil MFC.


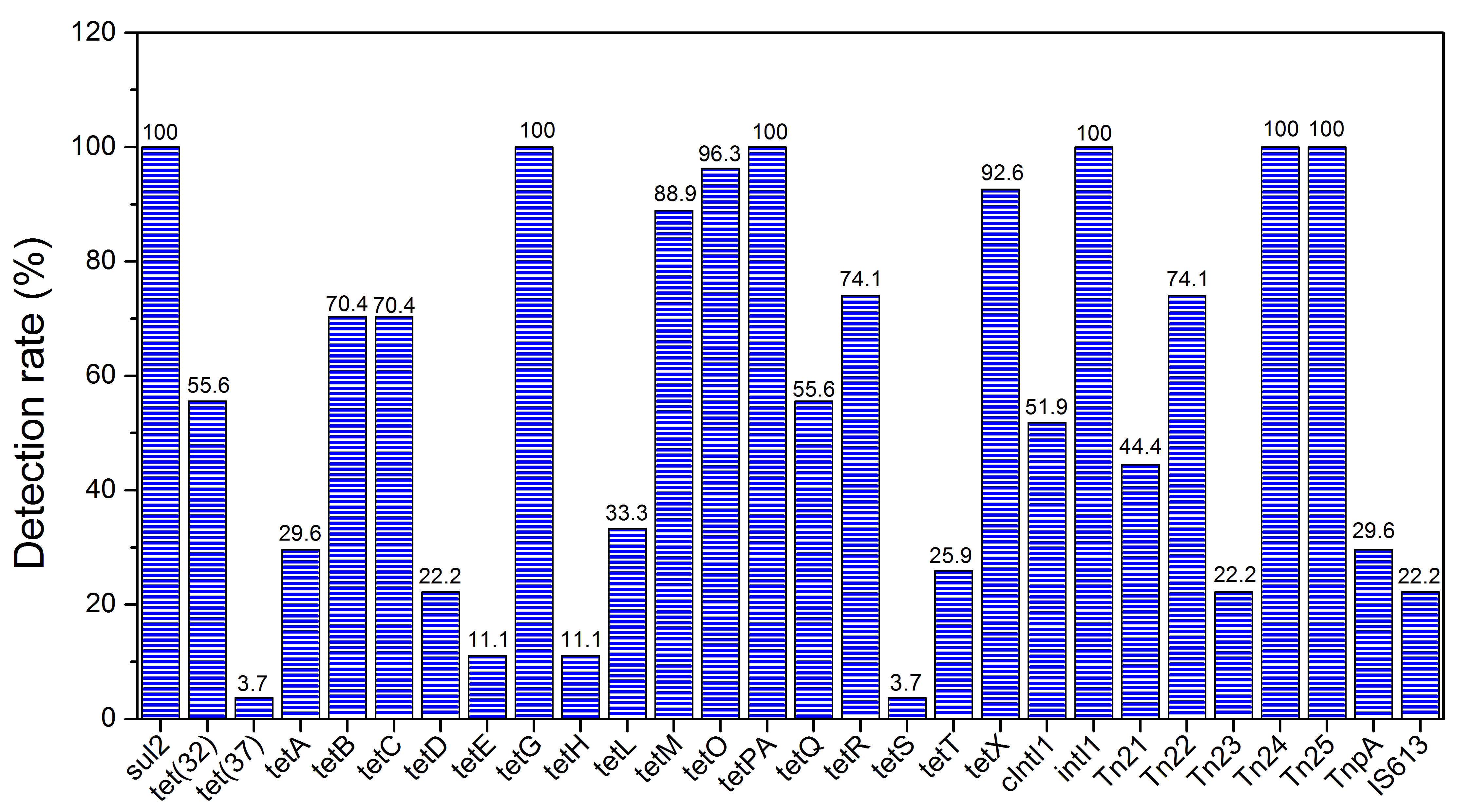


**Fig. S2.** Detection rates of ARGs (*tet* and *sul* genes) and MGE genes in tested soils (*n* = 27).

**
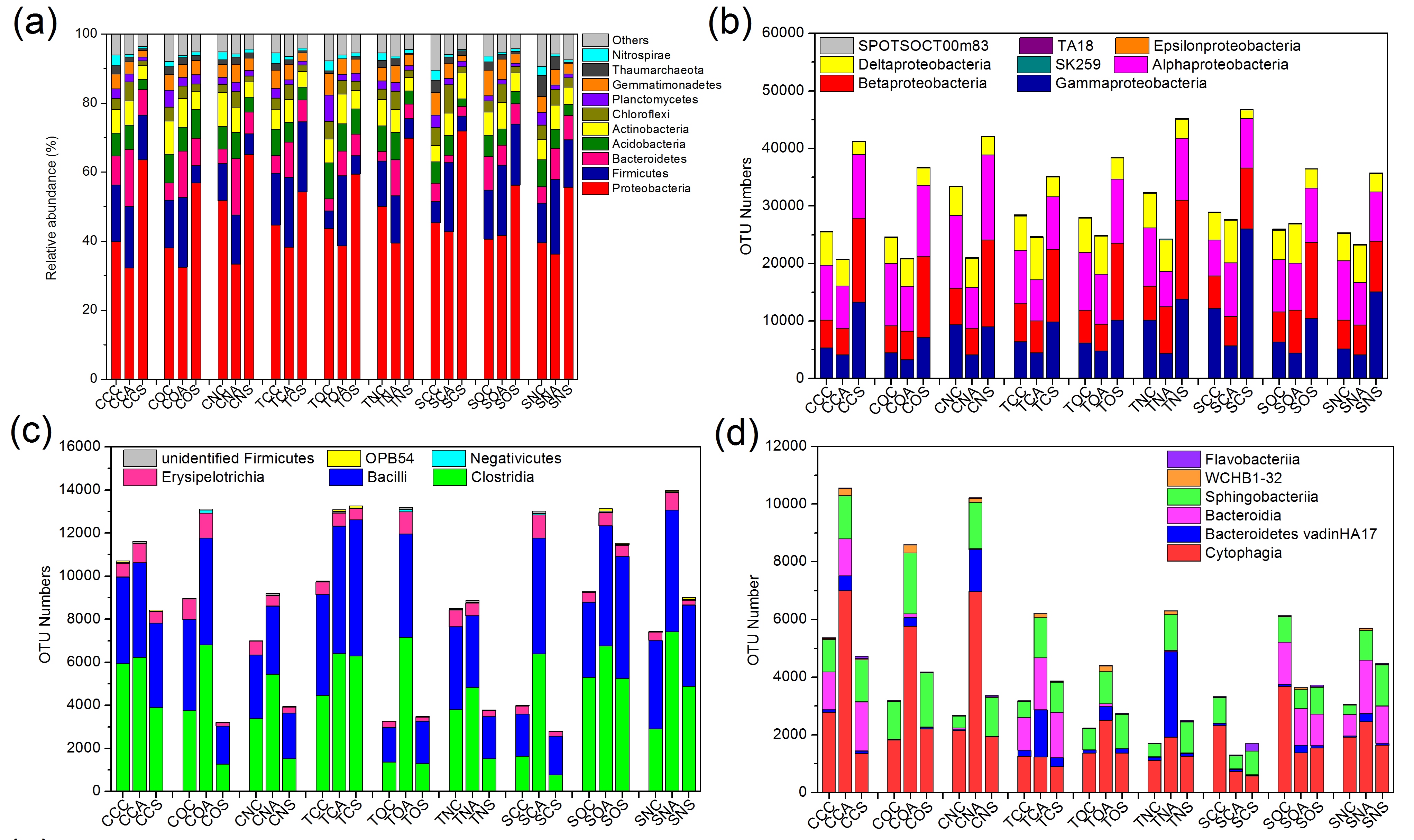
**

**Fig. S3.** Taxonomic classification of bacterial DNA sequences from soil communities in different layers of MFCs at the phylum level (a), the class level distribution of the dominant phyla of *Proteobacteria* (b), *Firmicutes* (c), *Bacteroides* (d). CC/CO/CN/TC/TO/TN/SC/SO/SNC, CC/CO/CN/TC/TO/TN/SC/SO/SNA and CC/CO/CN/TC/TO/TN/SC/SO/SNS represent layer C, layer A and layer S in the same rector, respectively.


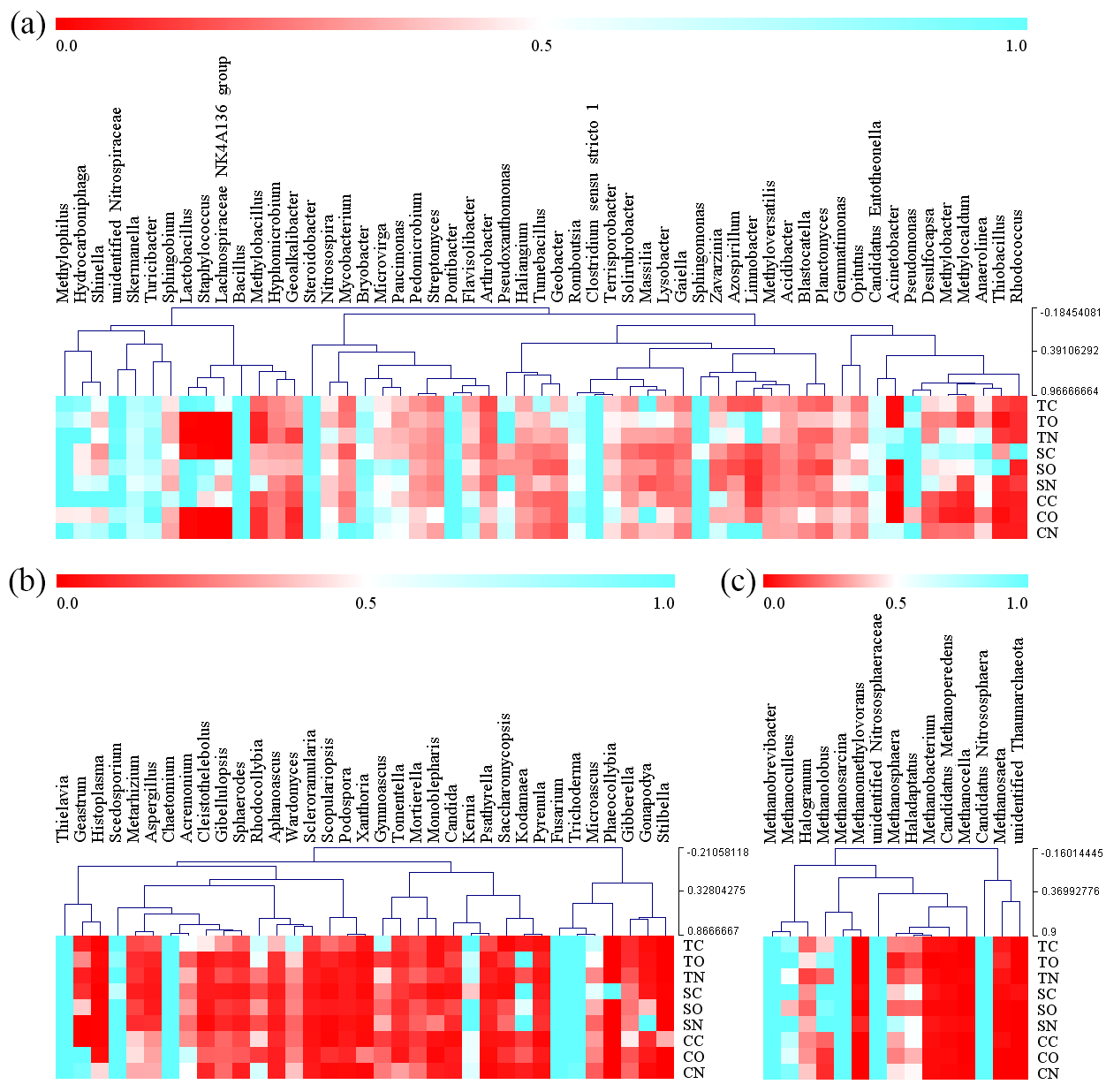


**Fig. S4.** Taxonomic classification of the microbial DNA sequences from soil communities in the MFCs at the genus level for bacteria (a), fungi (b), and archaea (c).


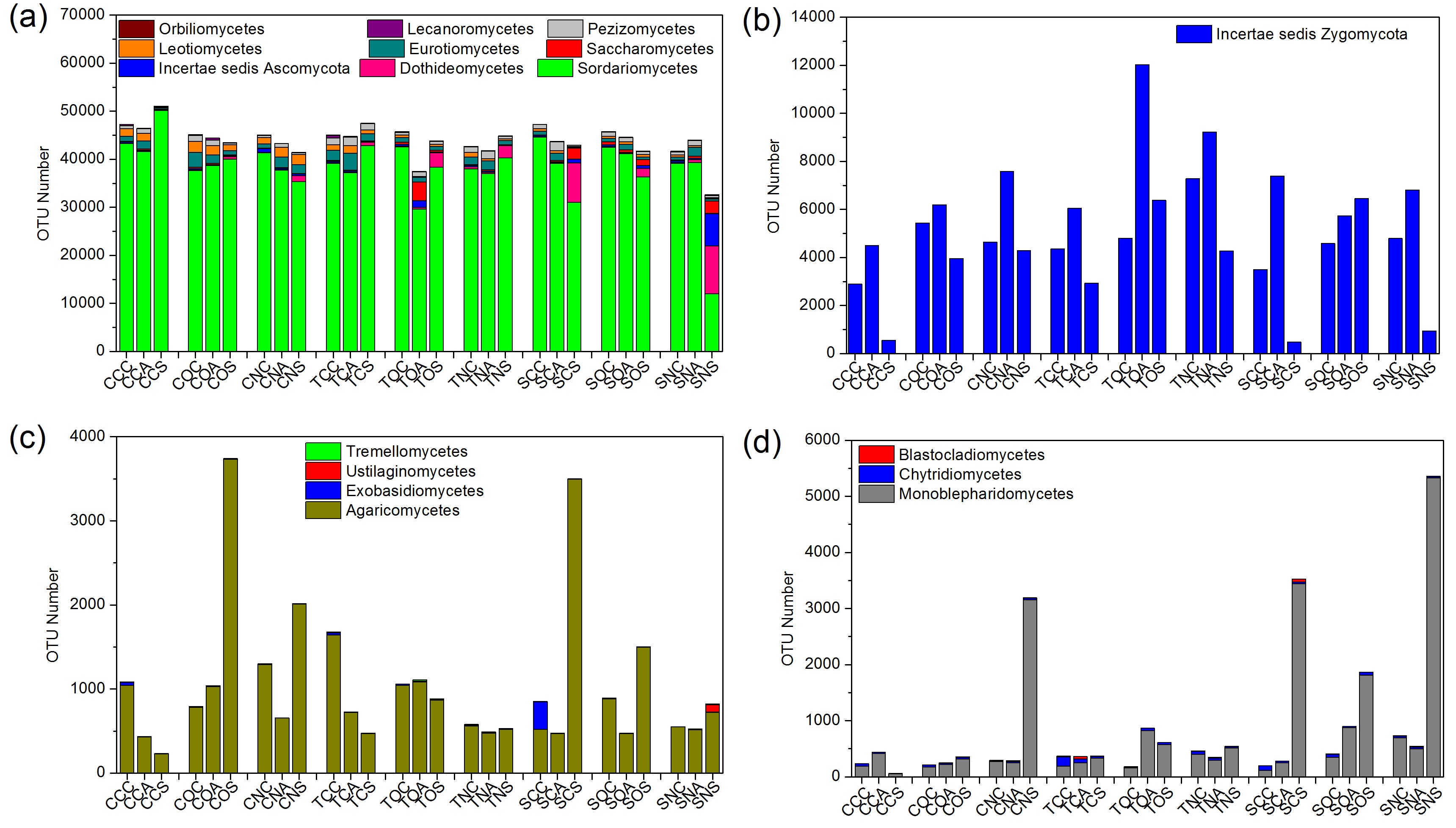


**Fig. S5.** Taxonomic classification of fungal DNA sequences from soil communities in different layers of MFCs the class level distribution of the dominant phyla of *Ascomycota* (a), *Zygomycota* (b), *Basidiomycota* (c), *Chytridiomycota* (d).


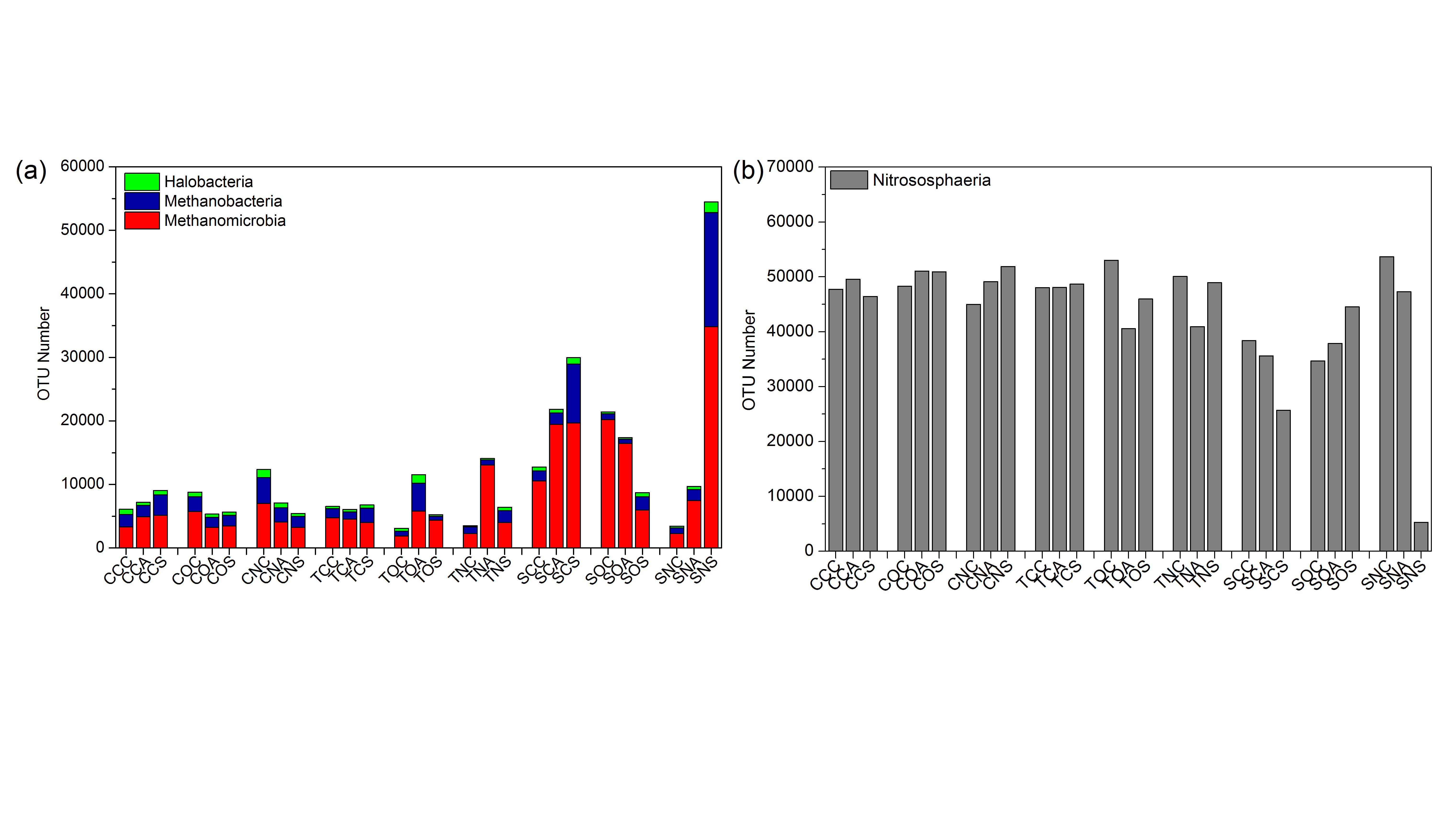


**Fig. S6.** Taxonomic classification of archaeal DNA sequences from soil communities in different layers of MFCs the class level distribution of the dominant phyla of *Euryarchaeota* (a), *Thaumarchaeota* (b).


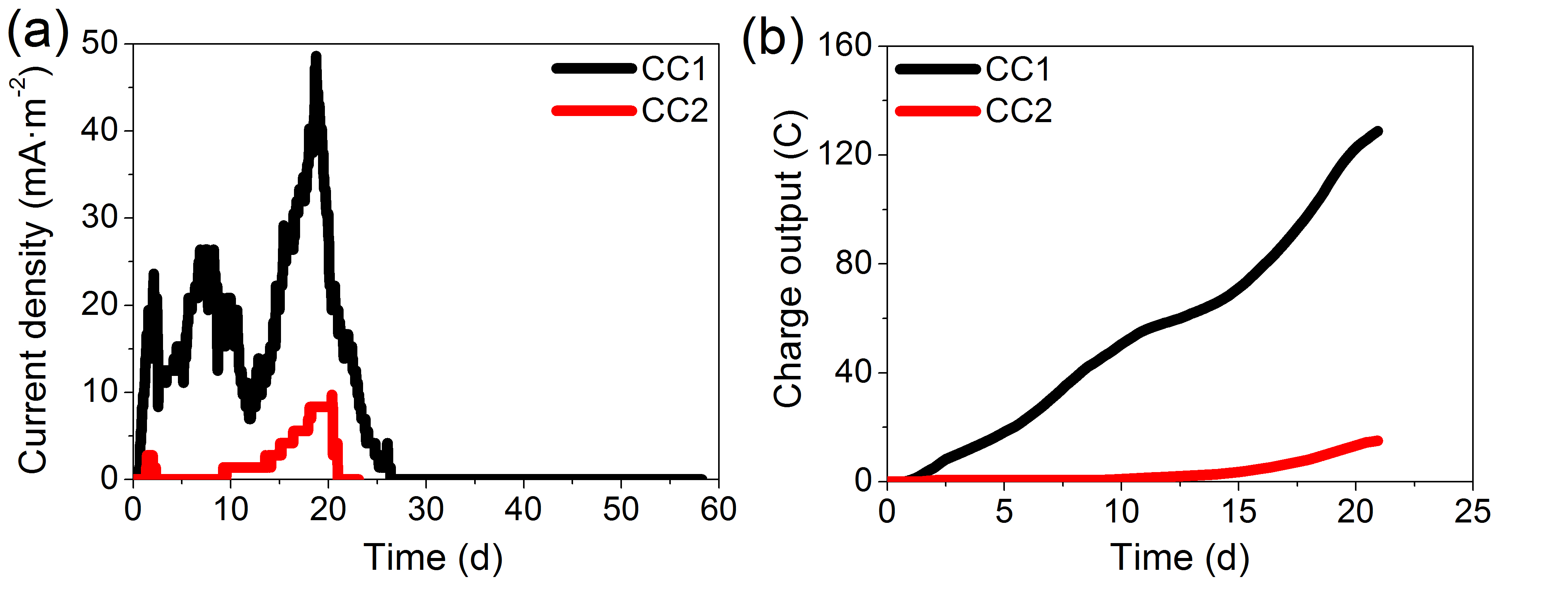


**Fig. S7.** Comparison of current densities (a) and charge output (b) of soil MFCs between CC1 (in our study) and CC2. *Cathodic microbial community adaptation to the removal of chlorinated herbicide in soil microbial fuel cells, Environmental Science and Pollution Research, 2018, 25(17), 16900-16912*.


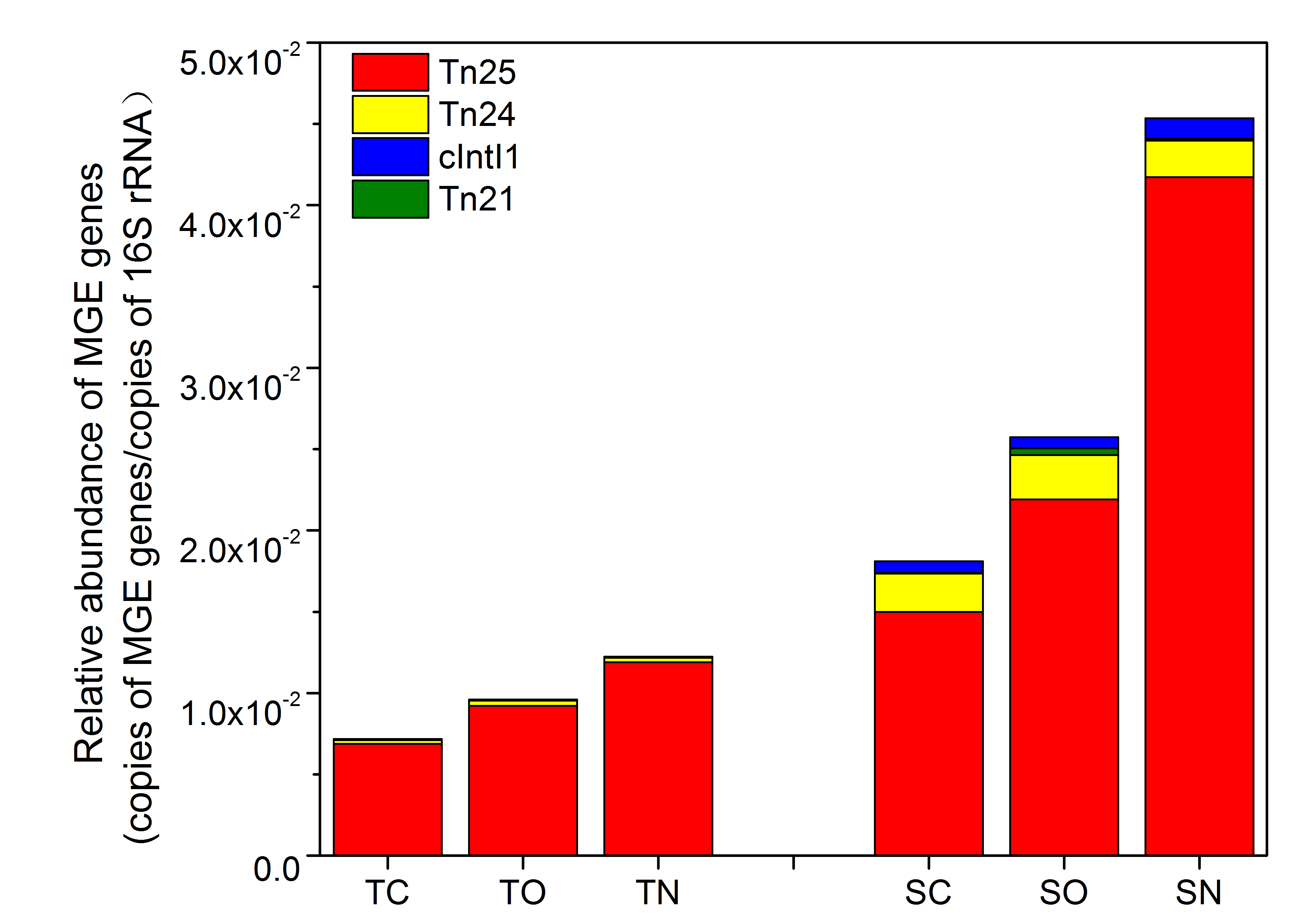


**Fig. S8.** The sum relative abundance of *Tn*21, *Tn*24, *Tn*25 and *cIntI*1 in treatments spiked with tetracycline and sulfadiazine.
